# Chloride regulates leaf cell size and water relations in tobacco plants

**DOI:** 10.1093/jxb/erv502

**Published:** 2015-11-23

**Authors:** Juan D. Franco-Navarro, Javier Brumós, Miguel A. Rosales, Paloma Cubero-Font, Manuel Talón, José M. Colmenero-Flores

**Affiliations:** ^1^Instituto de Recursos Naturales y Agrobiología, CSIC, Avda Reina Mercedes 10, 41012-Sevilla, Spain; ^2^Instituto Valenciano de Investigaciones Agrarias, Centro de Genómica, Ctra Moncada-Náquera Km 4.6, 46113-Moncada, Valencia, Spain

**Keywords:** Beneficial nutrient, chloride nutrition, growth, osmotic potential, turgor, water potential, water balance, water relations, water-use efficiency, WUE.

## Abstract

Chloride (Cl^–^) is a micronutrient that accumulates to macronutrient levels since it is normally available in nature and actively taken up by higher plants. Besides a role as an unspecific cell osmoticum, no clear biological roles have been explicitly associated with Cl^–^ when accumulated to macronutrient concentrations. To address this question, the glycophyte tobacco (*Nicotiana tabacum* L. var. Habana) has been treated with a basal nutrient solution supplemented with one of three salt combinations containing the same cationic balance: Cl^–^-based (CL), nitrate-based (N), and sulphate+phosphate-based (SP) treatments. Under non-saline conditions (up to 5mM Cl^–^) and no water limitation, Cl^–^ specifically stimulated higher leaf cell size and led to a moderate increase of plant fresh and dry biomass mainly due to higher shoot expansion. When applied in the 1–5mM range, Cl^–^ played specific roles in regulating leaf osmotic potential and turgor, allowing plants to improve leaf water balance parameters. In addition, Cl^–^ also altered water relations at the whole-plant level through reduction of plant transpiration. This was a consequence of a lower stomatal conductance, which resulted in lower water loss and greater photosynthetic and integrated water-use efficiency. In contrast to Cl^–^, these effects were not observed for essential anionic macronutrients such as nitrate, sulphate, and phosphate. We propose that the abundant uptake and accumulation of Cl^–^ responds to adaptive functions improving water homeostasis in higher plants.

## Highlight

Chloride is actively taken up and accumulated to macronutrient levels in higher plants, leading to adaptive functions that improve growth and water relations, acting as a beneficial macronutrient.

## Introduction

Chloride (Cl^–^) is one of the 16 elements essential for plant growth. Because it is supposedly needed in small quantities for healthy growth of plants (<50–100 μM in the nutrient media), Cl^–^ is classified as a micronutrient (Johnson *et al.*, 1957; Whitehead, 1985). Usually, in non-halophytic plants, the critical deficiency concentration is <0.2mg g^-1^ shoot DW (reviewed in Flowers, 1988; Xu *et al.*, 2000; White and Broadley, 2001; Broadley *et al.*, 2012a). Under this critical threshold, plants tend to show a significant decrease in leaf area as a result of a reduction in cell division rates (Terry, 1977). As an essential micronutrient, Cl^–^ is involved in the stabilization of the water splitting system of photosystem II (PSII) and the regulation of enzyme activities such as the asparagine synthethase, amylases, and the tonoplast H^+^-ATPase. Important Cl^–^ functions are also related to the electrical charge balance of essential cations such as K^+^ and H^+^, playing main roles in the stabilization of the electric potential of cell membranes and the regulation of pH gradients (reviewed in Xu *et al.*, 2000; White and Broadley, 2001; Hänsch and Mendel, 2009; Broadley *et al.*, 2012a). In addition, Cl^–^ is an osmotically active solute in the vacuole. Participation of Cl^–^ in cell osmotic regulation has been debated largely through its involvement in the regulation of cell turgor processes such as stomatal movement and the activity of motor cells controlling nastic movements (reviewed in Flowers, 1988; White and Broadley, 2001; Broadley *et al.*, 2012a). Preferential accumulation of Cl^–^ has been reported in epidermal cells of barley (Leigh and Tomos, 1993) and epidermal cells from elongating internodes of *Pisum sativum* (Yamagami *et al.*, 2004). Since higher plants are thought to perform normally with low Cl^–^ content, it is generally accepted that specific osmoregulatory functions of Cl^–^ are confined to these tissues, where Cl^–^ content should be higher than the average of the bulk tissues (Flowers, 1988).

However Cl^–^ does not appear to be a typical micronutrient since the actual Cl^–^ concentration in plants, in the range of 2–20mg g^–1^ DW (Xu *et al.*, 2000; Brumós *et al.*, 2010; Broadley *et al.*, 2012a), is 10–100 times higher than the concentration required as an essential micronutrient. This is relevant provided that all other mineral micronutrients (B, Cu, Fe, Mn, Mo, Ni, and Zn) are present at much lower concentrations in plant tissues (10^–4^–10^–1^ mg g^-1^ DW), while the accumulation to higher levels results in plant toxicity (Hänsch and Mendel, 2009). Cl^–^ transport occurs primarily via the symplastic pathway (Pitman, 1982; Brumós *et al.*, 2010), and Cl^–^ uptake under non-saline conditions is an electrogenic Cl^–^/2H^+^ symport mechanism requiring metabolic energy (Felle, 1994; White and Broadley, 2001; Britto and Kronzucker, 2006). As Cl^–^ accumulation to macronutrient concentrations requires a considerable use of energy (Brumós *et al.*, 2010), it is feasible to think that Cl^–^ plays a broader and poorly understood biological role, one that is certainly not critical under normal growth conditions. Since Cl^–^ appears to be particularly well suited to accomplishing osmoregulatory roles, we wondered whether Cl^–^ accumulation at macronutrient concentrations is specifically involved in the regulation of water relations in plants at both the cell and the whole-plant level. It is generally assumed that, when accumulated in large quantities, Cl^–^ serves a non-specific osmotic function in the vacuole, where it is interchangeable with other solutes such as nitrate (NO_3_^–^; Fricke *et al.*, 1994). This is a controversial issue for several reasons: (i) usually the role of Cl^–^ is not adequately differentiated from that of their accompanying cations (reviewed in Flowers, 1988); (ii) or, very frequently, some physiological and molecular responses have been entirely attributed to the cation (e.g. K^+^) in experimental approaches where the effect of the accompanying anion (Cl^–^) has not been controlled (see, for instance, Armengaud *et al.*, 2004; Benlloch-Gonzalez *et al.*, 2008); (iii) it is unclear to what extent Cl^–^ is preferred by plants to fulfil osmoregulatory roles or whether other inorganic anions, such as the macronutrients NO_3_^–^, sulphate (SO_4_^2–^), or phosphate (PO_4_^3–^) can replace Cl^–^ in such functions; and (iv) the idea linking Cl^–^ homeostasis with osmotic/turgor regulation has been frequently discussed in the context of salt stress and halophyte species (Yeo and Flowers, 1980, 1986; Flowers and Yeo, 1986; Flowers, 1988; Perez-Perez *et al.*, 2007). For instance, Cl^–^ was frequently assumed to move passively in plants, resulting in toxic effects in leaves of some woody species such as Citrus under high or moderate salt stress (Moya *et al.*, 1999; Storey and Walker, 1999; Moya *et al.*, 2003). We have reported that under non-saline conditions Cl^–^ is actively taken up and accumulated into leaf tissues of Citrus plants to levels that exceed the critical content requirement by one order of magnitude in the so-called Cl^–^-excluder rootstocks and by two orders in Cl^–^-includer (salt-sensitive) rootstocks, through symplastically regulated transport mechanisms (Tadeo *et al.*, 2008; Brumos *et al.*, 2010). This is indicative that Citrus (and probably other glycophyte plants) handle Cl^–^ homeostasis similarly to a macronutrient such as K^+^ rather than a toxic ion such as Na^+^.

In this work, it is our aim to establish the specific role of Cl^–^ in glycophyte plants when accumulated to macronutrient levels, and it is shown here that Cl^–^ nutrition in the millimolar range (1–5mM) specifically stimulates growth and increased leaf cell size, regulating leaf tissue water balance and water relations in tobacco plants.

## Materials and methods

### Experimental design

Tobacco plants (*Nicotiana tabacum* L. var. Habana) were grown under greenhouse conditions at 24±2 °C/17±2 °C (day/night), a relative humidity of 60±10% (EL-1-USB Data-logger, Lascar Electronics Inc., Erie, PA, USA), and a 16h/8h photoperiod with a photosynthetic photon flux density (average PAR) of 300–350 µmol m^–2^ s^–1^ (quantum sensor, LI-6 400; Li-COR, Lincoln, NE, USA) and a luminous emittance of 9000–10 000 lux (Digital Lux Meter, LX1010B; Carson Electronics, Valemount, Canada). Tobacco seeds were sown in flat trays (cell size 4 cm×4 cm×10cm) containing peat that had been previously washed with the corresponding nutrient solution. After vernalization for 2 d in a cold chamber (4 °C), seeds were transferred to the greenhouse. After 3 weeks, seedlings were transplanted to 7.5 litre pots (pot size 20 cm×17 cm×25cm), containing a mix of perlite:coarse sand:vermiculite (2:3:5), where plants were watered through a semi-hydroponics regime with the different nutrient solutions described below.

In Johnson *et al.* (1957), 50 μM Cl^–^ was established as the treatment ensuring Cl^–^ micronutrient requirements in different plant species, whereas deficiency symptoms were obtained in plants with no Cl^–^ addition. In this study, 75 μM Cl^–^ (added as CoCl_2_ 11 μM+KCl 53 μM) was chosen to be always present in the basal nutrient solution (BS) to fulfil plant requirements in low Cl^–^ treatments, but not to allow significant Cl^–^ accumulation in plant tissues. This Cl^–^ concentration was confirmed through direct measurement of the Cl^–^ concentration in the BS medium using a chloridometer (see below). Other nutrients present in the BS solution were: KNO_3_ 1.25mM, KH_2_PO_4_ 0.725mM, K_2_HPO_4_ 0.073mM, Ca(NO_3_)_2_ 2mM, MgSO_4_ 1mM, FeNa-EDTA 0.1mM, H_3_BO_3_ 0.1mM, MnSO_4_ 0.1mM, ZnSO_4_ 29 µM, CoCl_2_ 0.11 µM, KCl 53 μM, CuSO_4_ 0.1 µM, Na_2_MoO_4_ 1 μM, and 5 μM KI.

This work contains results from five independent experiments (see details in Supplementary Table S1 avalable at *JXB* online): January–February 2012 (JF2012); March–April 2012 (MA2012); November–December 2012 (ND2012); April–May 2013; (AM2013); and September–October 2013 (SO2013). Most experiments (JF2012, MA2012, ND2012, and SO2013) were performed with the application of 5mM Cl^–^ (CL treatment), which included the following salt mixtures supplemented in the BS solution: 2.5mM KCl, 0.625mM MgCl_2_, and 0.625mM CaCl_2_. In order to evaluate the specificity of Cl^–^ in the studied phenomena, two additional treatments were used: 5mM nitrate (NO_3_^–^) treatment (N) containing 2.5mM KNO_3_, 0.625mM Mg(NO_3_)_2_, and 0.625mM Ca(NO_3_)_2_; and sulphate+phosphate (SO_4_^2–^+PO_4_^3–^) treatment (SP) containing 1.25mM KH_2_PO_4_, 0.625mM K_2_SO_4_, 0.625mM MgSO_4_, and 0.625mM CaSO_4_. The various combinations of nutrient salts used, containing the same cationic balance, are listed in Supplementary Table S2. For the AM2013 experiment, increasing concentrations of anions were used in CL treatments: 0mM Cl^–^ (BS treatment containing 0.075mM Cl^–^), 0.15mM Cl^–^, 0.30mM Cl^–^, 1.0mM Cl^–^, 2.5mM Cl^–^, or 5.0mM Cl^–^; and the equivalent SP treatments ensuring the same cationic balance as in the different CL treatments (Supplementary Table S2). All experimental solutions were adjusted to pH 5.7 with KOH.

Pots were irrigated up to field capacity (3.5ml g^-1^ substrate) throughout the experiment. Pots were weighed each week at field capacity to estimate indirectly the increase of plant FW over time. After 6–7 weeks (65–72 d after sowing, DAS), shoots were harvested, FW values were obtained, and samples were dried in a forced-air oven at 75 °C for 48h to obtain the DW. Roots were rinsed with tap water and subsequently with distilled water and, after removing the excess water with filter paper, FW measurements were obtained. DW was obtained as for shoot harvesting. Dry tissues were ground to powder using a homogenizer (Taurus, 25 790 Barcelona, Spain), for subsequent analyses.

### Nutrient content determination

Fully photosynthetic and expanded mature leaves (non-senescent) from plants of 65–72 DAS were ground to powder as previously described. For Cl^–^ content determination, powdered leaf tissue was incubated overnight in a 0.1M HNO_3_ and 10% glacial acetic acid solution. After centrifuging, 0.5ml of the extract was used for the determination of Cl^–^ concentration in a Corning 926 chloridometer (Sherwood Scientific Ltd, Cambridge, UK) by silver ion titration according to the manufacturer’s instructions and to Gilliam (1971). Other soluble macro- and micronutrients were extracted in water from powdered dry tissues: the NO_3_^–^ concentration was measured through a multiparameter ‘Bran+Luebbe’ autoanalyser (Bran+Luebbe Analytics, Norderstedt, Germany); SO_4_^2–^ and PO_4_^3–^ concentrations were measured as described in Novozamsky and Van Eck (1977), and Hogue *et al.* (1970), respectively; and cation content was determined through inductively coupled plasma optical emission spectrometry (ICP-OES) using a Varian ICP 720-ES spectrometer (Varian Inc., Palo Alto, CA, USA).

### Leaf area

Detached leaves were scanned in an Epson Stylus DX4 000 multifunction printer (Seiko Epson Corp., Owa, Japan). Scanning settings were defined as: colourless b/w and a very low resolution (72 ppp). Leaf area was measured through pixel quantification with the ADN Software ‘Medición de Hojas v1.0’ (Developed at the Department of Ecology, University of Seville, Spain; Taguas and Rivero, 1989). Data were obtained in cm^2^. Specific leaf area (SLA) was calculated as follows (Marcelis *et al.*, 1998):

$$SLA=\left(Total\;leaf\;area\right)\;{\left(Total\;leaf\;DW\right)}^{-1}$$ (1)

### Water parameters and photosynthesis

Water content (WC, Equation 2), relative water content (RWC, Equation 3), and succulence (Equation 4) were determined in leaves obtained from 4–6 plants, using 3–4 leaves per plant and 10 discs per leaf (1cm diameter), and calculated as follows (Barrs and Weatherley, 1962):

$$WC\;\left(\%\right)=[\left(100\times \left(FW-DW\right)\right]\;{\left(FW\right)}^{-1}$$ (2)

$$RWC\;\left(\%\right)=\left(FW-DW\right)\;{\left(TW-DW\right)}^{-1}$$ (3)

$$Succulence\;\left(g\;cm^{-2}\right)=\left(FW-DW\right)\;{\left(leaf\;area\right)}^{-1}$$ (4)

where DW is the dry weight, FW is the fresh weight, and TW is the fully hydrated weight of the leaf. For TW calculation, the petiole of the whole leaf was imbibed in water for 4h before measuring the fresh weight of the leaf at full turgor.

Leaf osmotic potential (Ψ_π_) was calculated from the leaf sap obtained from 360 leaf discs: 20 leaf discs per sample×three samples per plant×six plants per treatment. Leaf sap was extracted from leaf discs by transferring the samples, placed in 0.5ml microcentrifuge tubes, from a block heated to 90 °C to liquid nitrogen. Tube caps were sealed with parafilm to avoid water evaporation. This thermal shock was repeated five times and leaf sap was collected in 1.5ml microcentrifuge tubes by centrifugation and filtration of tissue debris. Leaf water potential (Ψ_w_) was directly obtained from a fresh leaf disc (6–8 disc measurements per plant and six plants per treatment). Both parameters, Ψ_π_ and Ψ_w_, were recorded using the dew-point microvoltimeter (model HR-33T, Wescor, UT, USA) and the C-52 sample chamber as previously described in Cochrane (1994) and Colmenero-Flores *et al.* (1999). Before measuring, samples were incubated in the chambers for 40min to reach water vapour equilibration. Leaf turgor (or pressure) potential (Ψ_p_) was calculated from the Ψ_w_ and Ψ_π_ values (experimentally obtained) according to Equation 5:

$$\Psi_{p}(MPa)=|\Psi_{\pi }\left|-\right|\Psi_{w}|$$ (5)

Water consumption was quantified by measuring the volume of the semi-hydroponics nutrient solution consumed by the plants. Integrated water-use efficiency (WUE) was calculated as the increase of plant FW over time related to the accumulated water consumption (g FW ml H_2_O^–1^), as well as the final FW obtained after harvesting related to total water consumption (g FW ml H_2_O^–1^) (Abbate *et al.*, 2004).

Leaf gas exchange measurements were conducted between 12:00h and 14:00h using a gas-exchange system (LI-6 400: LI-COR, Lincoln, NE, USA). For each treatment, three photosynthetically active and fully expanded intermediate leaves from 4–6 plants (52–62 DAS) were used. Photosynthesis was induced with ambient light and 400 μmol mol^–1^ CO_2_ surrounding the leaf. Leaf temperature was maintained at 25 °C, and the leaf-to-air vapour pressure deficit was kept between 1 kPa and 1.3 kPa. These conditions were kept constant for the determinations of the net photosynthetic rate (*A*_N_; μmol CO_2_ m^–2^ s^–1^) and stomatal conductance (*g*_s_; mol H_2_O m^–2^ s^–1^). The intrinsic water-use efficiency (WUE) was calculated as the ratio between the photosynthetic rate and stomatal conductance (A_*N*_/*g*_s_; μmol CO_2_ mol^–1^ H_2_O; Rosales *et al.*, 2012). For the AM2013 experiment, which consisted of growing plants in anion concentration treatments, *g*_s_ was measured using the Decagon Leaf Porometer (Decagon Devices Inc., Pullman, WA, USA).

Assay of the leaf transpiration in an individual detached leaf, also called fresh weight loss (FWL), was carried out as previously described (Fernández *et al.*, 1997; Cheong *et al.*, 2007; Li *et al.*, 2014) with some modifications. FWL was calculated according to Equation 6 as the percentage of fresh weight loss over time upon leaf detachment:

$$FWL\;\left(\%\right)=\left(100\times LWC_{t}{}_{=x}\right){\left(LWC_{t}{}_{=0}\right)}^{-1}$$ (6)

where LWC_*t*_ is the leaf water content at time ‘*t*=*x*’ or at time zero ‘*t*=0’.

Leaves of plants at 65 DAS were detached and dehydrated over a filter paper at 24±2 °C, a relative humidity of 60±10%, and a photosynthetic photon flux density of 300 µmol m^–2^ s^–1^. Measurements of FW were recorded 0, 0.5, 1, 1.5, 2.5, 3, and 6h after leaf separation. Six plants per treatment and three detached leaves per plant were used.

Using the above FWL values, the percentage of saved water in treated plants (SP, N, and CL) was obtained according to Equation 7 and Equation 8 by subtracting the percentage of water loss of BS plants 6h after detachment. Water saving at the whole-plant level was calculated using integrated WUE values obtained by gravimetric measurement as described previously and according to Equation 9 and Equation 10 by subtracting the percentage of water loss of treated plants from the percentage of water loss of BS plants.

Water loss (Transpiration, %)=100−FWL (%) (7)

$$\begin{array}{l} Water\;saving\;\left(Transpiration,\;\%\right)=\\ Water\;loss\;BS\left(\%\right)-Water\;loss\;treated\;\left(\%\right) \end{array}$$ (8)

$$\begin{array}{l} Water\;loss\;\left(Consume,\;\%\right)=\\ \left(100\times WUE\;treated,\;\%\right)\;{\left(WUE\;BS,\;\%\right)}^{-1} \end{array}$$ (9)

$$\begin{array}{l} Water\;saving\;\left(Consume,\;\%\right)=\\ 100-Water\;loss\;\left(Consume,\;\%\right) \end{array}$$ (10)

The PSII quantum yield (QY) is a plant stress marker that quantifies the PSII efficiency. Chlorophyll fluorescence in light-adapted plants was measured using a portable fluorometer (FluorPen FP-100; Photon System Instruments, Brno, Czech Republic). The PSII QY in light-adapted plants (Equation 11) was calculated according to Maxwell and Johnson (2000) through the measurement of the following variable parameters (Equation 12). For each determination, four readings were taken from each leaf and averaged; three leaves from each plant and six plants per treatment were monitored every day.

$$QY=\Phi_{PSII}=F_{m}\prime {\left(F_{v}\prime \right)}^{-1}$$ (11)

$$F_{v}\prime =\left(F\prime_{m}-F_{t}\right)$$ (12)

where *F*_v_′ is the difference between *F*_m_′ (the maximum fluorescence in the light-adapted state) and *F*_t_ (the basal fluorescence in the light-adapted state).

### Anatomical parameters

To measure leaf thickness, histological preparations of tobacco leaves were produced as previously described in Scafaro *et al.* (2011). Analysis of abaxial leaf cells was carried out in epidermal peels and epidermal impressions. Epidermal impressions were performed as described in Gitz and Baker (2009). Peelings were carried out as described in He *et al.* (2013) and Allen *et al.* (1999), but with slight differences: peels were obtained from the abaxial surface by gently ripping the epidermis with a scalpel and removing it with tweezers. Peels were transferred to incubation buffer (10mM MES-KOH pH 6.5, 10mM KCl, and 50 µM CaCl_2_) before analysis by light microscopy. Histological preparations of leaf tissue were made to analyse mesophyll cells (spongy and palisade cells). Leaf tissues were fixed overnight in FAA (4% formaldehyde, 5% acetic acid, and 50% ethanol), dehydrated through a graded ethanol series, and then embedded in Paraplast Plus (Sigma-Aldrich, http://www.sigmaaldrich.com) as described previously (Langdale, 1994). Microtome sections (8 µm) were stained with Safranin/Fast Green (Sigma-Aldrich). Examination of epidermal peels, epidermal impressions, and histological preparations was performed on a Zeiss Axioskop microscope equipped with Nomarski optics, AxioCam MRc5, and the Zeiss AxioVision software (Freeware ‘Carl Zeiss AxioVision Rel.4.9.1.0’ available at the Zeiss Homepage http://www.zeiss.com/, Carl Zeiss Microscopy GmbH, Jena, Germany). Cell size was determined as either the cross-sectional area of mesophyll cells or the cell surface measured from epidermal impressions in epidermal cells. Cell count was performed using the Counterall^*®*^ software (www.counterall.com, Bioscripts.net-IRNAS-CSIC). The total number of epidermal cells per leaf was estimated according to the Equation 13:

$$\begin{array}{l} Total\;no.\;of\;epidermal\;cells\;per\;leaf=\;\\ \left(Epidermal\;cell\;frequency\right)\times Total\;leaf\;area\;\left(cm^{2}\right) \end{array}$$ (13)

Where epidermal cell frequency is the total number of epidermal cells per cm^2^. Measurement of cell size was performed using the outline tool of the AxioVision Software.

### Statistical analysis

Statistical analysis was performed using the STATGRAPHICS Centurion XVI software (http://www.statgraphics.com; StatPoint Technologies, Warrenton, VA, USA). Shapiro–Wilk test was used to verify the normality of the data sets. One-way ANOVA and multivariate analysis of variance (MANOVA) were performed to determine significant differences between groups of samples, and levels of significance are indicated in the figures by asterisks: *P≤0.05; **P≤0.01; ***P≤0.001. Non-significant (ns) differences were indicated when *P*>0.05. Multiple comparisons of means were determined by the Tukey’s HSD (honestly significant difference) and MRT (multiple range test) statistical tests included in the above-mentioned software. Values represent the mean of 4–6 tobacco plants in each treatment. Correlations between logarithm of anion concentration and physiological parameters were calculated through the Pearson’s product–moment correlation coefficient (*R* ^2^).

## Results

**Fig. 1.**
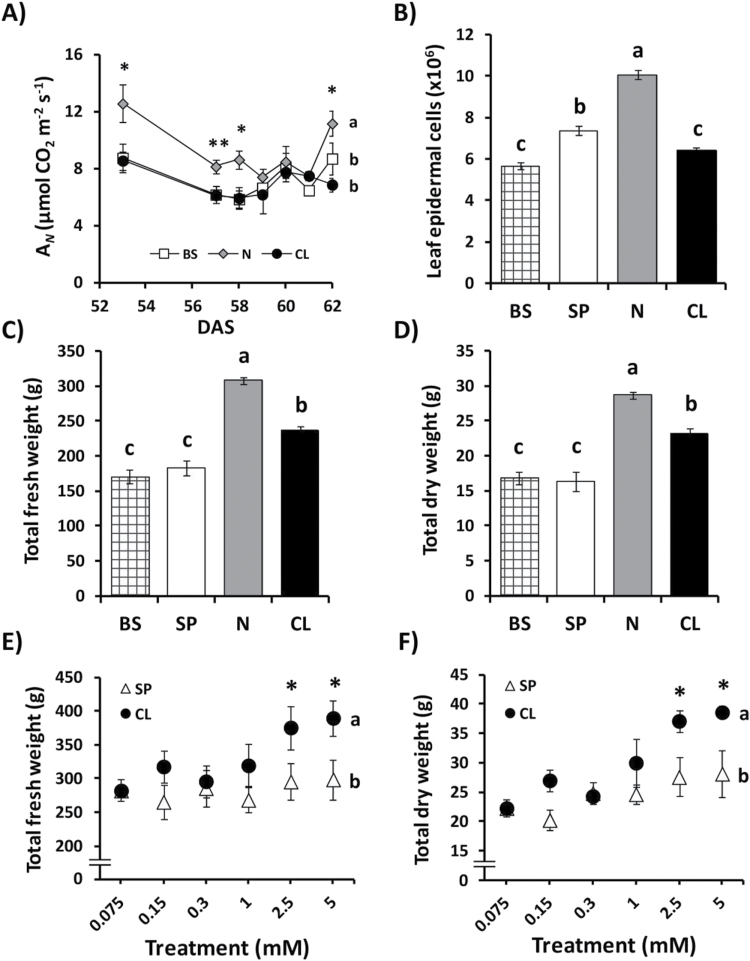
Effect of Cl^–^ nutrition on photosynthesis, cell division, and growth. In (A–D), treatments consisted of the application of the basal nutrient solution (BS) alone or supplemented with 5mM chloride (CL), 5mM nitrate (N), or the sulphate+phosphate (SP) salt mixture containing the same cationic balance as in the CL and N treatments. (A) Effect on net photosynthetic rate (*A*_N_) measured in fully expanded, photosynthetically active leaves from tobacco plants between 53 d and 62 d after sowing (DAS). (B) Effect on cell division rate, quantified as the number of epidermal cells per leaf (abaxial side). (C, D) Effect on total (shoot and roots) FW and DW at the end of the experiment. (E, F) Effect of increasing concentrations of Cl^–^ (CL) or sulphate+phosphate (SP) treatments, maintaining the same cationic balance, on total (shoot and roots) FW and DW, respectively. A non-linear scale is used on the horizontal axis for dose–response experiments (E and F). Mean values ± SE, *n*=6. Levels of significance: *P≤0.05; **P≤0.01. ‘Homogeneous group’ statistics were calculated through ANOVA (A–F) and MANOVA (A, E, F) tests.

We initially studied the effects of Cl^–^ application in the millimolar range (CL treatment) on plant Cl^–^ content and growth, and we also verified that plants subjected to low Cl^–^ treatments (BS, N, and SP) were not experiencing Cl^–^ deficiency. The leaf cation concentration (K^+^, Ca^2+^, Mg^2+^) was similar in plants treated with CL, N, and SP supplements, whereas anions were differentially accumulated according to the respective treatment (Table 1; Supplementary Table S3 at *JXB* online). The Cl^–^ concentration in CL-treated plants was similar to the concentration of the macronutrient K^+^, and significantly higher than the NO_3_^–^ concentration in N-treated plants or the SO_4_^2–^+PO_4_^3–^ concentration in SP-treated plants. Comparing the anion concentration in CL versus N and SP treatments, it was observed that the molar excess of Cl^–^ over NO_3_^–^ was 2.30, and that of Cl^–^ over SO_4_^2–^+PO_4_^3–^ was 3.05, indicating that Cl^–^ (not assimilated) was preferentially accumulated over other anionic macronutrients (which are assimilated) (Table 1). Cl^–^ was preferentially accumulated in leaves, with a molar excess of 4.2-fold over root Cl^–^ concentration and 3.2-fold over stem Cl^–^ concentration (Supplementary Table S4). Consistent with the intracellular accumulation of negative charges of biomolecules, a higher concentration of inorganic cations was observed in all treatments (Tables 2, 3). However, the contribution of inorganic anions to equimolarity with inorganic cations (Table 2) or to electrical neutrality (Table 3) was higher in CL-treated plants. This indicates that CL plants require less accumulation of organic molecules (e.g. organic acids) involved in osmoregulation or electric neutrality processes (Tables 2, 3). The Cl^–^ concentration in leaves of plants treated with low Cl^–^ (BS, SP, and N treatments) exceeded by 3–5 times the threshold of Cl^–^ deficiency (Supplementary Fig. S1), and no deficiency symptoms such as wilting, chlorosis, or bronzing were observed (not shown). Cl^–^ is an essential cofactor in photosynthesis, although the primary aspect involved in the reduced growth under Cl^–^ deficiency was a lower rate of cell division in the leaves (Terry, 1977). We verified that photosynthesis and leaf cell division rates were not impaired in plants subjected to low Cl^–^ treatments (BS, SP, or N). The highest photosynthetic activity (Fig. 1A; Supplementary Figs S4B, S5A) and the highest number of epidermal leaf cells (Fig. 1B) were observed in N-treated plants. Since the lowest Cl^–^ content was always observed in N and SP plants (Supplementary Fig. S1), we concluded that low Cl^–^ treatments covered essential Cl^–^ requirements. Although Cl^–^ is thought to be a micronutrient, tobacco plants responded positively to 5mM Cl^–^ application in terms of FW (Fig. 1C) and DW (Fig. 1D) when compared with BS or SP treatments. As expected, N-treated plants showed the highest growth and biomass because of the important role of nitrogen in plant growth and development (Hawkesford *et al.*, 2012). Although biomass varied depending on the season in which the experiments were conducted, the CL treatment always determined a significant biomass increase compared with BS and SP treatments (Supplementary Fig. S2). The biomass increase determined by the CL treatment required Cl^–^ application at >1mM external concentration (Fig. 1E, F), ~50–100 times the external concentration required as a micronutrient (Supplementary Table S5). Taken together, these results show that Cl^–^ in the millimolar range stimulates plant growth, and ruled out the possibility that plants subjected to low Cl^–^ treatments were experiencing Cl^–^ deficiency.

**Table 1.** Ion concentration in leaves subjected to different treatments

Treatment	Ion concentration (mmol g^-1^ DW)						
	K^+^	Ca^2+^	Mg^2+^	Cl^–^	NO_3_^–^	PO_4_^3–^	SO_4_^2–^
BS	0.905±0.047 b	0.391±0.029	0.252±0.021	0.028±0.003 b	0.255±0.061 b	0.128±0.002 a	0.126±0.022 b
SP	1.238±0.059 a	0.355±0.033	0.336±0.046	0.015±0.002 b	0.144±0.028 bc	0.136±0.080 a	0.336±0.039 a
N	1.259±0.060 a	0.465±0.016	0.305±0.033	0.017±0.002 b	0.627±0.102 a	0.081±0.006 b	0.105±0.005 b
CL	1.268±0.047 a	0.381 ±0.054	0.317 ±0.032	1.441±0.061 a	0.039±0.007 c	0.104±0.004 b	0.111±0.008 b
*P*-value	***	ns	ns	***	***	***	***

Treatment consisted of the basal nutrient solution (BS) supplemented with 5mM chloride (CL), 5mM nitrate (N), or the sulphate+phosphate (SP) salt mixture containing the same cationic balance as in the CL and N treatments.

Mean values ± SE, *n*=6. Levels of significance: *P*>0.05 (‘ns’, non-significant differences); ***P≤0.001). ‘Homogeneous group’ statistics were calculated through ANOVA test.

**Table 2.** Contribution of inorganic anions to equimolarity with cations in the leaf

	Ionic concentration (mmol g^–1^ DW)		Equimolarity (%)		Contribution to equimolarity (%)		
	Cations	Anions	Inorganic anions	Organic molecules^*a*^	SO_4_^2–^+PO_4_^3–^	NO^–^	Cl^–^
BS	1.76±0.05 b	0.58±0.09 b	32.6±4.4 b	67.4±4.4 a	14.3±0.9 b	17.1±3.4 a	1.1±0.2 b
SP	1.96±0.08 ab	0.65±0.08 b	32.7±3.6 b	67.3±3.6 a	24.3±1.9 a	7.7±2.2 b	0.7±0.05 b
N	2.10±0.05 a	0.72±0.06 b	34.4±3.0 b	65.6±3.0 a	8.9±0.3 b	25.0±2.9 a	0.5±0.02 b
CL	2.02±0.07 a	1.49±0.04 a	74.3±2.0 a	25.7±2.0 b	10.6±0.4 b	0.8±0.1 b	62.9±2.0 a
*P*-value	*	***	***	***	***	***	***

Treatments consisted of the basal nutrient solution (BS) alone or supplemented with 5mM nitrate (N) or the sulphate+phosphate (SP) salt mixture containing the same cationic balance as in the CL and N treatments.

Anions and cations measured to calculate the data in the respective columns were: Cl^–^, NO_3_^–^, SO_4_^2–^, and PO_4_^3–^anions; and K^+^, Ca^2+^, and Mg^2+^ cations.

Mean values ± SE, *n*=4–6. Levels of significance: *P≤0.05 and ***P<0.001. ‘Homogeneous group’ statistics was calculated through ANOVA test.

^*a*^ Not measured: deduced from quantification of inorganic ions.

**Table 3.** Contribution of inorganic anions to electrical neutrality with cations in the leaf

	Ionic concentration (mEq l^–1^)		Contribution to electrical neutrality (%)		Contribution of individual anions to electrical neutrality (%)		
	Positive charges of inorganic cations	Negative charges of inorganic anions	Inorganic anions	Organic molecules^*a*^	SO_4_^2–^+PO_4_^3–^	NO_3_^–^	Cl^–^
BS	205.83±7.12	81.48±8.53 b	40.6±5.0 b	59.3±5.0 a	26.6±2.0 b	13.1±3.0 ab	0.9±0.2 b
SP	216.10±8.33	108.66±13.48 b	49.1±4.1 b	50.9±4.1 a	42.6±2.8 a	6.0±1.7 bc	0.5±0.04 b
N	208.51±1.75	75.11±5.33 b	35.9±2.3 b	64.0±2.3 a	16.5±0.6 c	19.0±2.0 a	0.4±0.02 b
CL	211.47±6.60	146.69±4.71 a	69.9±2.8 a	30.1±2.8 b	20.3±0.9 bc	1.0±0.3 c	48.5±2.0 a
*P*-value	ns	***	***	***	***	***	***

Treatments consisted of the basal nutrient solution (BS) alone or supplemented with 5mM nitrate (N) or the sulphate+phosphate (SP) salt mixture containing the same cationic balance as in the CL and N treatments.

Anions and cations measured to calculate the data in the respective columns were: Cl^–^, NO_3_^–^, SO_4_^2–^, and PO_4_^3–^anions; and K^+^, Ca^2+^, and Mg^2+^ cations.

Mean values ± SE, *n*=4–6. Levels of significance: *P*>0.05 (‘ns’, non-significant differences) and ***P<0.001. ‘Homogeneous group’ statistics were calculated through ANOVA test.

^*a*^ Not measured: deduced from quantification of inorganic ions.

**Fig. 2.**
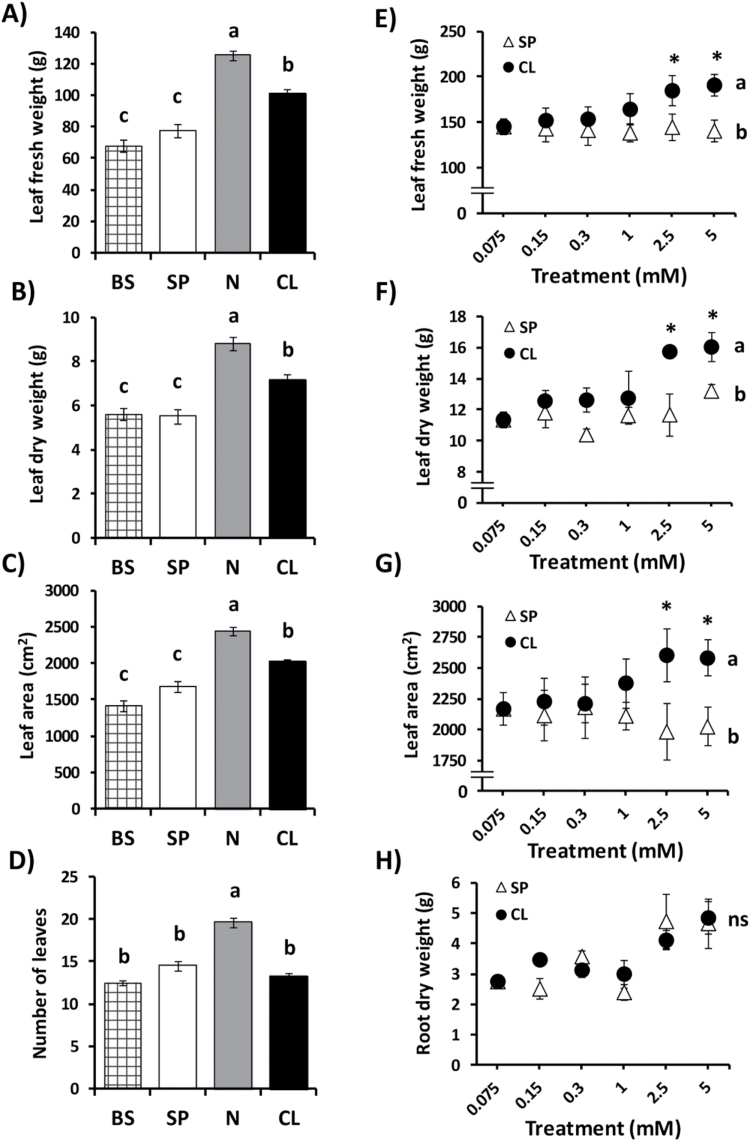
Effect of Cl^–^ nutrition on leaf growth parameters. In (A–D), treatments consisted of the application of the basal nutrient solution (BS) alone or supplemented with 5mM chloride (CL), 5mM nitrate (N), or the sulphate+phosphate (SP) salt mixture containing the same cationic balance as in the CL and N treatments. In (E–H), treatments consisted of increasing concentrations of Cl^–^ (CL) or sulphate+phosphate (SP) salts maintaining the same cationic balance. (A and E) Effect on total leaf FW. (B and F) Effect on total leaf DW. (C and G) Effect on total leaf area. (D) Effect on number of leaves. (H) Effect on root DW. A non-linear scale used on the horizontal axis for dose–response experiments (E–H). Mean values ± SE, *n*=6. Levels of significance: *P* > 0.05 (‘ns’, non-significant differences); *P≤0.05; and ‘homogeneous group’ statistics were calculated through ANOVA (A–H) and MANOVA (E–H) tests.

**Fig. 3.**
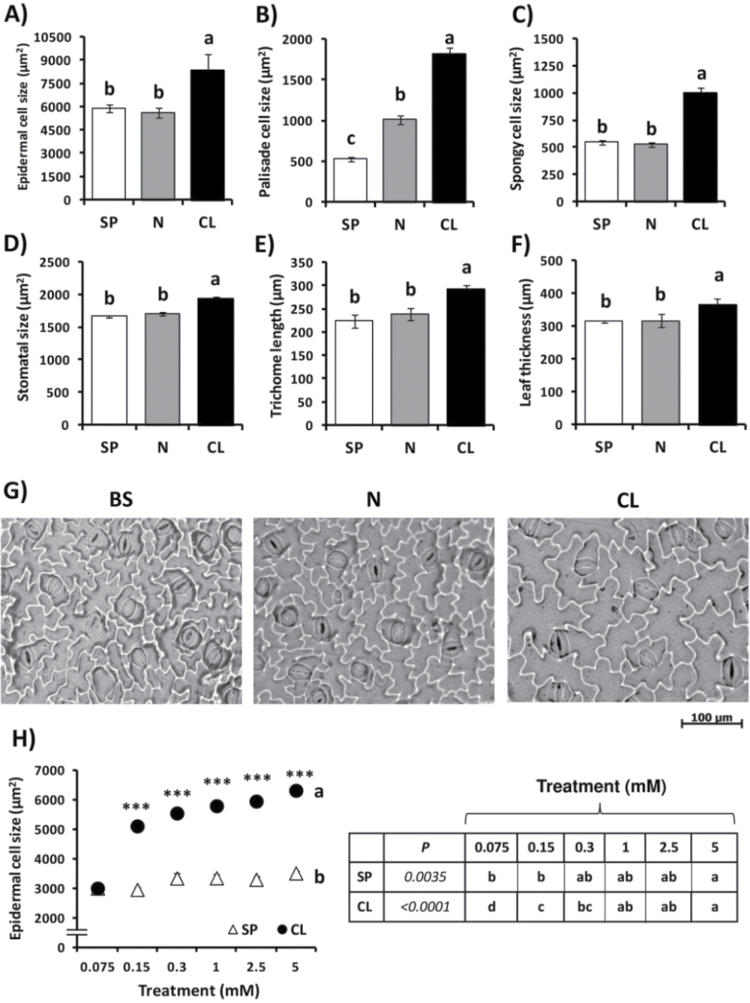
Effect of Cl^–^ nutrition on leaf cell size. In (A–G), treatments consisted of the application of the basal nutrient solution (BS) alone or supplemented with 5mM chloride (CL), 5mM nitrate (N), or the sulphate + phosphate (SP) salt mixture containing the same cationic balance as in the CL and N treatments. In (H), treatments consisted of increasing concentrations of Cl^–^ (CL) or sulphate+phosphate (SP), maintaining the same cationic balance. (A, H) Effect on epidermal cell size. (B) Effect on palisade mesophyll cell size. (C) Effect on spongy mesophyll cell size. (D) Effect on stomatal size (combined surface area of the two guard cells and the pore space between). (E) Effect on trichome length. (F) Effect on leaf thickness. (G) Effect of the treatments BS, N, and CL on epidermal cell size observed in microscopy images obtained from abaxial leaf epidermal impressions. Epidermal and stomatal size values were obtained from abaxial epidermal imprints; mesophyll cells size and leaf thickness values were obtained from transversal sections; and trichome length was directly measured from epidermal peel preparations. A non-linear scale is used on the horizontal axis for the dose–response experiment (H). Mean values ± SE, *n*=6. Levels of significance: ***P≤0.001; and ‘homogeneous group’ statistics were calculated through ANOVA (A–F, H) and MANOVA (H) tests.

Compared with the BS and SP treatments, the CL treatment stimulated growth of shoot organs (stems and leaves), but not of the root (see Supplementary Fig. S3 at *JXB* online). The CL treatment stimulated total leaf FW (Fig. 2A), DW (Fig. 2B), and total leaf area (Fig. 2C), but not the number of leaves, which was stimulated only with the N treatment (Fig. 2D). Again, these growth parameters were increased with Cl^–^ treatments >1mM concentration (Fig. 2E–G), whereas no differences between SP and CL treatments were observed in root growth (Fig. 2H). The CL treatment specifically stimulated cell size of different leaf cell types including epidermal cells, mesophyll cells, guard cells, and trichomes (Fig. 3). This was particularly evident in epidermal cells (Fig. 3A, G; Supplementary Fig. S5F) and mesophyll cells (Fig. 3B, C), which were significantly more expanded in Cl^–^-treated plants. In addition, the CL treatment determined higher leaf thickness (Fig. 3F). Compared with the basal solution, containing 75 μM Cl^–^ (giving rise to 2.49mM leaf Cl^–^ concentration; Supplementary Table S5), strong cell growth stimulation was observed with a Cl^–^ treatment as low as 150 μM (giving rise to 6.55mM leaf Cl^–^ concentration; Supplementary Table S5), and this effect was progressively enhanced with increasing concentrations of Cl^–^ (Fig. 3H).

**Fig. 4.**
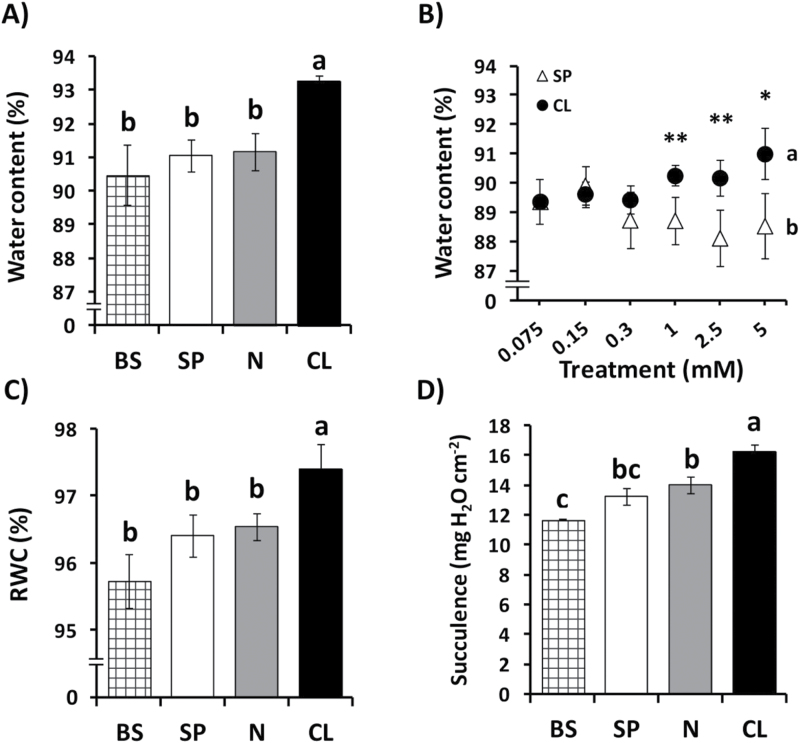
Effect of Cl^–^ nutrition on leaf water parameters. In (A and C–E), treatments consisted of the basal nutrient solution (BS) alone or supplemented with 5mM chloride (CL), 5mM nitrate (N), or the sulphate+phosphate (SP) salt mixture containing the same cationic balance as in the CL and N treatments. In (B), treatments consisted of increasing concentrations of Cl^–^ (CL) or sulphate+phosphate (SP), maintaining the same cationic balance. (A, B) Effect on leaf water content. (C) Effect on leaf relative water content (RWC). (D) Effect on leaf succulence. A non-linear scale is used on the horizontal axis for the dose–response experiment (B). Mean values ± SE, *n*=6. Levels of significance: *P ≤ 0.05; **P≤0.01; and ‘homogeneous group’ statistics were calculated through ANOVA (A-–E) and MANOVA (B) tests.

**Fig. 5.**
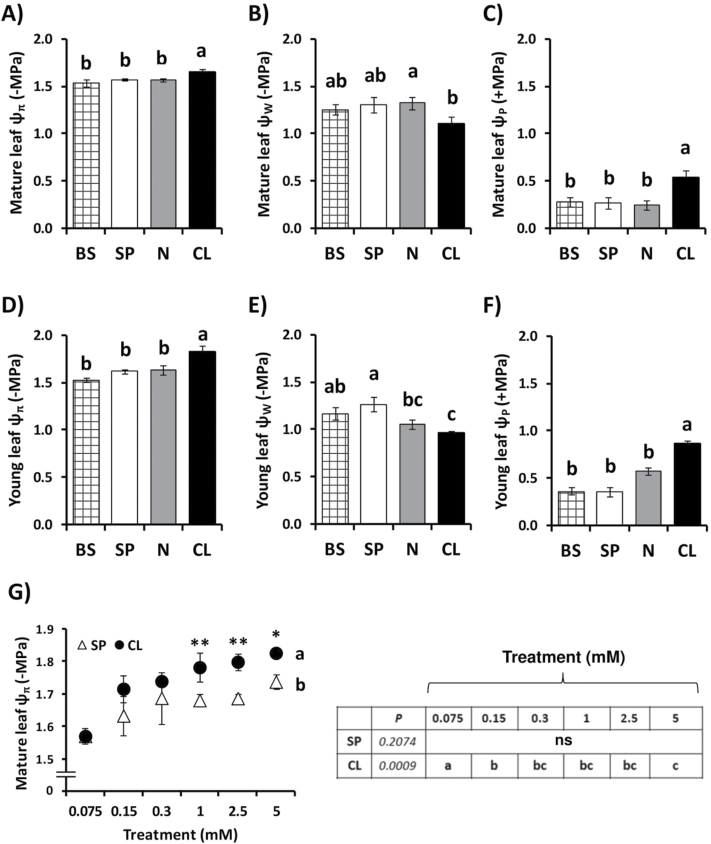
Effect of Cl^–^ nutrition on leaf osmotic potential, water potential, and estimated turgor. In (A–F), treatments consisted of the basal nutrient solution (BS) alone or supplemented with 5mM chloride (CL), 5mM nitrate (N), or the sulphate+phosphate (SP) salt mixture containing the same cationic balance as in the CL and N treatments. In (G), treatments consisted of increasing concentrations of Cl^–^ (CL) or sulphate+phosphate (SP) salts maintaining the same cationic balance. (A–G) Effect on leaf osmotic potential (ψ_π_), water potential (ψ_w_), and turgor (ψ_p_) measured using discs from mature (fully expanded) and young leaves (still expanding), harvested before dawn. A non-linear scale is used on the horizontal axis for the dose–response experiment (G). Values ± SE, *n*=6. Levels of significance: *P*>0.05 (‘ns’, non-significant differences); *P≤0.05; **P≤0.01; and ‘homogeneous group’ statistics were calculated through ANOVA (A–G) and MANOVA (G) tests.

**Fig. 6.**
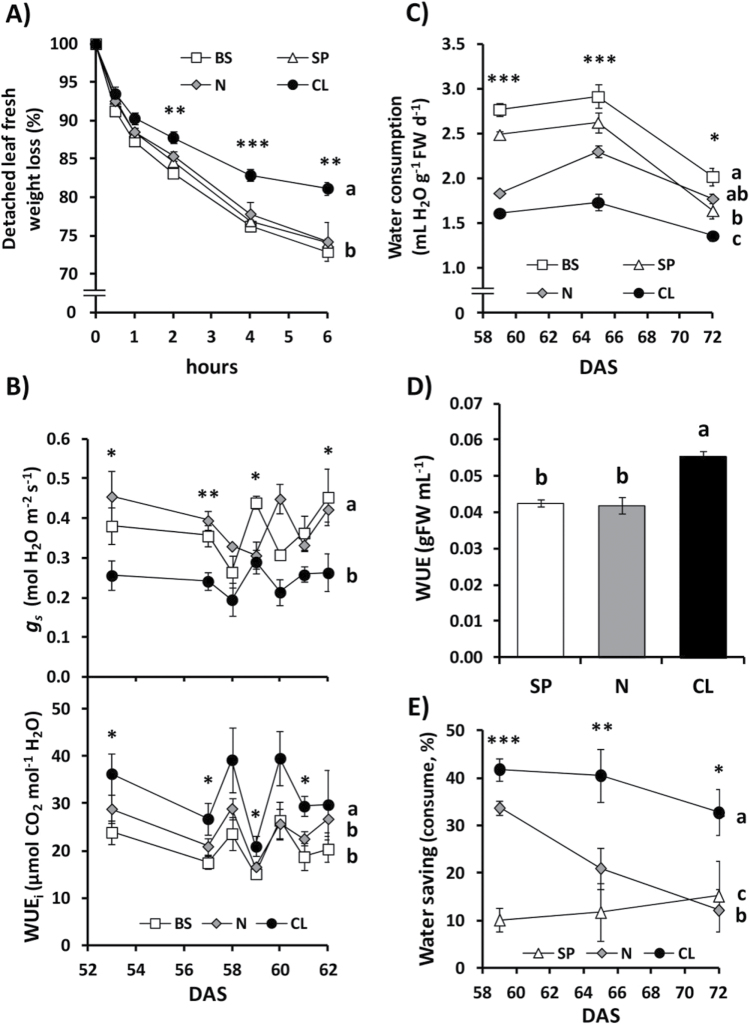
Effect of Cl^–^ nutrition on stomatal conductance, leaf-level water loss, whole-plant water use, and water-use efficiency. Treatments consisted of the basal nutrient solution (BS) alone or supplemented with 5mM chloride (CL), 5mM nitrate (N), or the sulphate+phosphate (SP) salt mixture containing the same cationic balance as in the CL and N treatments. (A) Effect on leaf transpiration measured as the FW loss of detached leaves over time. (B) Effect on stomatal conductance (*g*_s_) and photosynthetic or instantaneous water-use efficiency (WUE_i_) measured from plants between 53 d and 62 d after sowing (DAS). WUEi was calculated from both *A*_N_ data presented in Fig. 1A and *g*_s_ data presented in Fig. 6B. (C) Effect on total water consumed relative to the plant FW measured at different times after sowing and on each occasion over a 24h period. (D) Effect on integrated WUE obtained from total biomass produced in relation to total water consumed. (E) Effect on plant water saving capacity calculated as the water consumption (mL H_2_O g^–1^ FW d^–1^) of treated plants relative to the water consumption of BS plants (from data of (C) measured from plants between 58 and 72 DAS. Mean values ± SE, *n* = 4-6. Levels of significance: *P≤0.05; **P≤0.01; ***P≤0.001; and ‘homogeneous group’ statistics were calculated through ANOVA (A–E) and MANOVA (A–C, E) tests.

Our working hypothesis was that Cl^–^ nutrition in the millimolar range might specifically improve the hydric state of plant tissues and water parameters at the whole-plant level. In the leaf, water content (Fig. 4A), RWC (Fig. 4C), and succulence (Fig. 4D) were specifically stimulated by Cl^–^ over SP and N treatments. An increase of leaf water content required Cl^–^ application in the millimolar range (Fig. 4B). To determine whether higher water content was associated with higher osmotic capacity of Cl^–^-treated plants, leaf osmotic potential (Ψ_π_) and leaf water potential (Ψ_w_) values were measured. We confirmed that Ψ_π_ was more negative in both mature and young leaves of plants treated with Cl^–^, indicating a higher amount of osmotically active solutes in their tissues (Fig. 5A, D). Interestingly, leaf Ψ_w_ of CL-treated plants was less negative than leaf Ψ_w_ measured in plants subjected to BS, SP, and N treatments (Fig. 6B, E). Leaf turgor (Ψ_p_) of CL plants was significantly higher than Ψ_p_ of BS, SP, and N plants (Fig. 5C, F). Treatments with increasing concentrations of both CL and SP salt supplements determined progressive reductions of leaf Ψ_π_, although the effect was stronger and the differences were significant with CL treatments in the millimolar range (Fig. 5G). The observation that leaf Ψ_w_ was less negative in CL plants compared with BS, SP, and N treatments indicated that besides the osmotic effect, other events occurred in leaves of CL plants, determining higher water accumulation. Water potential values of well-watered tobacco plants in the range of –0.9MPa to –1.3MPa may seem quite negative for a cultivated plant. The dew-point method to determine leaf Ψ_w_ is a well-accepted method yet is also well documented to lead easily to incorrect values in the lower MPa range. In any case, these values are within the range of Ψ_w_ values measured by others in well-irrigated *Solanaceae* species (see, for example, Vos and Oyarzún, 1987; Yelenosky and Guy, 1989; Chartzoulakis and Drosos, 1995; Smith and Hare, 2004). In addition, these measurements were made in plants with leaf water content values in the range of 90–93% (Fig. 4A) and RWC values in the range of 95–98% (Fig. 4C), which correspond to non-stressed plants with well-hydrated leaf tissues.

**Fig. 7.**
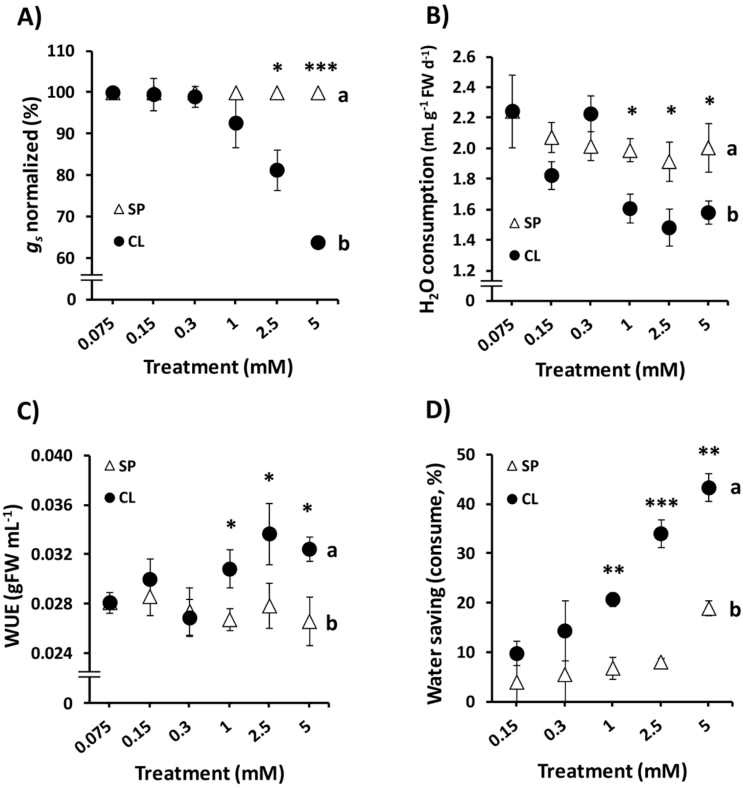
Effect of increasing concentrations of anions on whole-plant water relations. Treatments consisted of increasing concentrations of Cl^–^ (CL) or sulphate+phosphate (SP) salts maintaining the same cationic balance. The results were obtained on days 41, 42, and 47 after sowing (DAS). (A) Effect on stomatal conductance (*g*_s_) normalized to the *g*_s_ activity of SP plants. (B) Effect on total water consumption relative to the FW of plants. (C) Effect on integrated water-use efficiency (WUE) obtained as total biomass produced in relation to total water consumed. (D) Effect on plant water saving capacity calculated as the water consumption (mL H_2_O g^–1^ FW d^–1^) of treated plants relative to the water consumption of BS plants. A non-linear scale is used on the horizontal axis for dose–response experiments. Mean values ± SE, *n*=6. Levels of significance: *P ≤ 0.05, **P ≤ 0.01, and ***P ≤ 0.001; and ‘homogeneous group’ statistics were calculated through ANOVA and MANOVA tests.

In plants, the most important factor regulating water content is leaf transpiration. Interestingly, CL plants showed lower transpiration than SP, N, and BS plants, quantified as the relative loss of FW measured in detached leaves (Fig. 6A). Reduced transpiration of CL plants was a consequence of the lower stomatal conductance (*g*_s_) observed in intact Cl^–^-treated plants (Fig. 6B; Supplementary Figs S4C, S5B at *JXB* online), which in turn determined a higher photosynthetic WUE (Fig. 6B; Supplementary Figs S4D, S5C). As a consequence, CL plants consumed less water (Fig. 6C) and had higher integrated WUE, measured as total plant weight relative to total water consumed (Fig. 6D; Supplementary Fig. S5D, S5E). The basal treatment (BS) always resulted in higher water consumption, which was reduced with the application of salt supplements (Fig. 6C). We defined the parameter water saving as the percentage of water saved by the SP, N, or CL treatments in relation to the water consumed in BS plants. We distinguished two different water saving parameters: water saving from whole-plant water consumption, and water saving from leaf transpiration (obtained from the FWL assay). The CL treatment showed the greatest ability to promote water saving from whole-plant water consumption (~40% of BS consumption), significantly higher than the water saving promoted by SP and N treatments (Fig. 6E). Furthermore, the water saving determined by the 2.5–5mM Cl^–^ treatments compared with the SP treatment (~25% water saving; Fig. 7D) was quite similar to the *g*_s_ reductions quantified in plants treated with 2.5–5mM Cl^–^ relative to the corresponding SP treatments (~20–36% *g*_s_ reduction; Fig. 7A). Reduction of *g*_s_ and water consumption, the increased WUE, and plant water saving required the application of Cl^–^ in the millimolar range (Fig. 7).

**Fig. 8.**
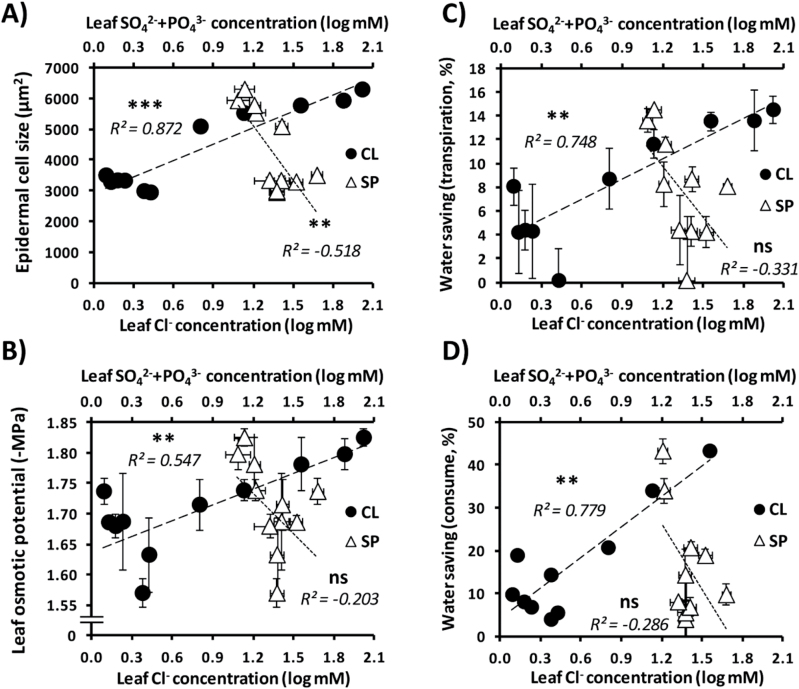
Correlations of leaf anion concentration with anatomical and plant water parameters. Treatments consisted of increasing concentrations of Cl^–^ (CL) or sulphate+phosphate (SP) salts maintaining the same cationic balance. Correlations with chloride concentration measured in both CL and SP plants are given with filled circles and correlations with sulphate+phosphate concentration measured in both CL and SP plants are given with open triangles. (A) Correlations with epidermal cell size. (B) Correlations with leaf osmotic potential. (C) Correlations with water saving capacity based on leaf transpiration measured as FW loss 6h after leaf detachment. (D) Correlations with water saving capacity calculated as the accumulated water consumption (mL H_2_O g^–1^ FW d^–1^) of treated plants relative to the accumulated water consumption of BS plants. Logarithms are in base 10. Mean values ± SE, *n*=6. Levels of significance represented by the Pearson’s *R* ^2^ linear correlation test and *P*>0.05 (‘ns’, non-significant differences); **P≤0.01; ***P≤0.001.

There are three important parameters identified in this work specifically associated with Cl^–^ treatments determining improved water homeostasis: leaf cell size, leaf osmotic potential, and water saving, obtained from both leaf transpiration and whole-plant water consumption data. These parameters were correlated to the internal leaf concentrations of Cl^–^ or SO_4_^2–^+PO_4_^3–^ obtained from plants treated with increasing concentrations of anions in CL and SP treatments, respectively. We found a positive correlation of leaf Cl^–^ concentration with these parameters, whereas no correlations were observed with SO_4_^2–^+PO_4_^3–^ concentration (Fig. 8).

Finally, to compare SP and CL treatments in plants with a similar internal leaf concentration of Cl^–^ and SO_4_^2–^+PO_4_^3–^ anions, different combinations of CL and SP treatments have been tested (see Supplementary Fig. S6 at *JXB* online). Using 1mM CL and 2.5mM SP treatments, we obtained comparable internal concentrations of Cl^–^ (35.82±1.52mM) and SO_4_^2–^+PO_4_^3–^ (33.65±3.72mM) anions, respectively. Also, using 1.83mM CL and 5mM SP treatments, we obtained comparable internal concentrations of Cl^–^ (55.46±7.50mM) and SO_4_^2–^+PO_4_^3–^ (48.01±4.25mM) anions, respectively. When these treatments were compared, we still observed increased capacity of Cl^–^ to promote the effects previously described herein (Tables 4, 5).

**Table 4.** Comparison of different water parameters in SP and CL plants containing similar concentrations of SO_4_^2–^+PO_4_^3–^ or Cl^–^ anions. respectively (~33mM)

	Leaf Ψ_π_ (–MPa)	Leaf WC (%)	Leaf succulence (mg H_2_O cm^–2^)	WUE (g FW ml^–1^)	Water consumed (mL H_2_O g FW^–1^ d^–1^)	Water saving (transpiration, %)	Water saving (consume, %)
SP 2.5 mM	1.68±0.02 b	88.1±0.9 b	14.34±0.68	0.026±0.001 b	1.91±0.13 a	4.6±1.7 b	8.1±0.8 b
CL 1 mM	1.79±0.02 a	90.2±0.3 a	15.47±0.66	0.032±0.001 a	1.54±0.07 b	12.4±1.6 a	19.2±2.0 a
*P*-value	*	*	ns	*	*	*	**

Different external concentrations of Cl^–^ (CL) or sulphate+phosphate (SP) salt supplements were applied to obtain plants with comparable leaf internal concentrations of Cl^–^ and SO_4_^2–^+PO_4_^3–^ anions (see Supplementary Fig. S6 at *JXB* online for more information). We distinguished two different water saving parameters: water saving calculated from whole-plant water consumption (measured gravimetrically) and water saving from leaf transpiration (obtained from the ‘detached fresh weight loss’ assay shown in Fig. 6A). Both parameters were calculated as the water loss of BS plants minus the water loss of treated plants.

Ψ_π_, leaf osmotic potential; WC, water content; WUE, water-use efficiency.

Mean values ± SE, *n*=4–6. Levels of significance: *P*>0.05 (‘ns’, non-significant differences), *P≤0.05 and **P<0.01. ‘Homogeneous group’ statistics were calculated through ANOVA test.

**Table 5.** Comparison of different water parameters in SP and CL plants containing similar concentration of SO_4_^2–^+PO_4_^3–^ or Cl^–^ anions. respectively (~50mM)

	Leaf Ψ_π_ (–MPa)	Leaf WC (%)	Leaf succulence (mg H_2_O cm^–2^)	WUE (g FW ml^–1^)	Water consumed (mL H_2_O g FW^–1^ d^-1^)	Water saving (transpiration, %)	Water saving (consume, %)
SP 5 mM	1.73±0.02 b	88.1±0.9 b	14.59±0.52 b	0.025±0.001 b	2.11±0.17 a	5.9±1.0 b	19.0±1.5 b
CL 1.83 mM	1.82±0.02 a	90.7±0.3 a	15.71±0.50 a	0.032±0.002 a	1.51±0.07 b	14.5±1.5 a	38.7±2.6 a
*P*-value	*	***	*	*	**	*	*

Different external concentrations of Cl^–^ (CL) or sulphate+phosphate (SP) salt supplements were applied to obtain plants with comparable leaf internal concentrations of Cl^–^ and SO_4_^2–^+PO_4_^3–^ anions (see Supplementary Fig. S6 at *JXB* online for more information). We distinguished two different water saving parameters: water saving calculated from whole-plant water consumption (measured gravimetrically) and water saving from leaf transpiration (obtained from the ‘detached fresh weight loss’ assay shown in Fig. 6A). Both parameters were calculated as the water loss of BS plants minus the water loss of treated plants.

Ψ_π_, leaf osmotic potential; WC, water content; WUE, water-use efficiency.

Mean values ± SE, *n*=4–6. Levels of significance: *P≤0.05, **P≤0.01, and ***P≤0.001. ‘Homogeneous group’ statistics were calculated through ANOVA test.

## Discussion

**Fig. 9.**
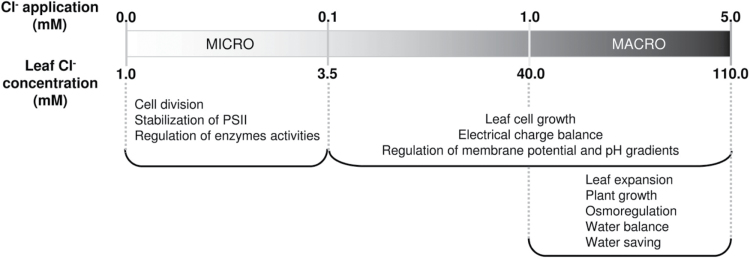
Schematic representation of Cl^–^ functions according to availability in the micro- or macronutrient range. Different biological functions are defined according to the external Cl^–^ application (0–5mM Cl^–^) or the resulting leaf bulk tissue concentration (1–110mM Cl^–^). Functions indicated in the 0.0–0.1mM Cl^–^ treatment range summarize previous knowledge of Cl^–^ roles as an essential micronutrient (see the Introduction); biological functions indicated in the 0.1–5.0mM Cl^–^ treatment range summarize the results obtained in this work.

Cl^–^ is ubiquitous in nature (typically found at concentrations of 1–5mM; Eaton, 1966) and easily available for plants (Broadley *et al.*, 2012a). Although it is described as a micronutrient, we show in this work that tobacco plants take up Cl^–^ to levels that are typical of the concentration of a macronutrient, playing specific biological roles that cannot be induced by *bona fide* macronutrient anions such as NO_3_^–^, SO_4_^2–^, and PO_4_^3–^. The most significant findings of this work are summarized in Fig. 9. When fed with Cl^–^ levels in the millimolar range (1–5mM), tobacco plants accumulate this anion in leaf tissues to high concentrations (40–110mM in the bulk leaf extract), promoting growth, leaf expansion, a better hydration state, reduced transpiration, higher WUE, and water saving. These acquired properties were associated with anatomical and physiological alterations in leaf tissues, which were also specifically triggered by Cl^–^, such as induction of larger cell size, lower osmotic potential, and higher cell turgor.

### Far from stress and deficiency conditions

In the context of plant biology, Cl^–^ has been generally associated with two extreme situations: its role as a micronutrient, required at low concentrations, and its effect on plants in salt stress conditions, due to excessive accumulation. It is important to note that the experimental conditions used in this work do not lead to any of these situations. In tobacco plants treated with 5mM Cl^–^, leaf Cl^–^ concentration was 51.08mg g^–1^ DW (1.44 mmol g^–1^ DW), comparable with the 49.56mg g^–1^ DW (1.27 mmol g^–1^ DW) potassium concentration measured in the same plants (Table 1; Supplementary Fig.S3 at *JXB* online). This value is similar to the concentration of Cl^–^ accumulated in leaves of Cl^–^-includer Citrus rootstocks treated with 4.5mM Cl^–^ for several months (Brumós *et al.*, 2010). These Cl^–^ treatments did not represent a stressful situation for plants in view of their normal appearance (not shown), normal growth (Figs 1, 2), and the normal performance of highly stress-sensitive parameters such as the stability of PSII (QY; *F*_m_′ *F*_v_′^–1^) and the plant photosynthetic rate (see Supplementary Fig. S4). To draw firm conclusions, it was very important to demonstrate also that under low Cl^–^ treatments no nutritional deficiency (as an essential micronutrient; Broadley *et al.*, 2012a) occurred in our control treatments. Unless elaborate precautions are taken to exclude all traces of chlorine/Cl^–^ from the moisture in the air and from the nutrient media, it is usually very difficult to induce Cl^–^ deficiency in plants (Johnson *et al.*, 1957; Whitehead, 1985). In this work, the minimal Cl^–^ treatment used was 75 μM, present in the basal nutrient solution, giving rise to leaf Cl^–^ concentrations >0.5mg g^–1^ DW (Supplementary Fig. S1; Supplementary Table S3), clearly exceeding the deficiency threshold of <0.2mg g^–1^ DW (Xu *et al.*, 2000; Broadley *et al.*, 2012a). Furthermore, the possibility that plants subjected to low Cl^–^ treatments were experiencing nutritional Cl^–^ deficiency was ruled out since: (i) BS, SP, and N plants, treated with low Cl^–^, did not exhibit visible deficiency symptoms; and (ii) N plants, normally containing half the concentration of Cl^–^ of BS plants (Supplementary Fig. S1; Supplementary Table S3), showed the highest performance in terms of photosynthetic activity, leaf cell division rates, and biomass (Fig. 1), which rules out a phenotype of Cl^–^ deficiency.

### Effect of Cl^–^ on plant biomass

As expected, plants supplemented with NO_3_^–^ exhibited the highest biomass as a consequence of the extra nitrogen fertilization giving rise to higher CO_2_ assimilation (Fig. 1A; Supplementary Figs S4A, S5A at *JXB* online) and strong stimulation of cell division and growth of all vegetative organs (Fig. 1B–D; Supplementary Figs S2, S3). However, in contrast to the stimulation of cell division observed in N plants, the biomass increase induced by Cl^–^ was associated with the stimulation of higher cell size and leaf growth (Figs 2, 3). Agronomic studies have reported a substantial increase in yield for many crops in response to Cl^–^ fertilization (reviewed in Xu *et al.*, 2000). It was not clear, however, to what extent this effect was due to the accompanying cations, or whether other anions could replace Cl^–^ in such a growth-promoting effect. The increase of biomass observed in this work (CL versus SP; Figs 1E, F, 2E–H; Supplementary Fig. S2) must be entirely attributed to Cl^–^ since the resulting internal concentration of cations in leaf tissues remained unchanged among the different treatments (Table 1; Supplementary Table S3). The difference in biomass was not due only to the increased water content of CL plants as its DW was also higher (Figs 1D–F, 2B–F; Supplementary Fig. S3), indicating that Cl^–^ treatments in the millimolar range also improved plant metabolism and growth.

The positive growth response to Cl^–^ was possibly related to a better hydration state of CL plants (Figs 4, 5 and 6), to more light interception of plants with greater leaf area (Fig 2C, G), and/or the idea previously suggested by Flowers (1988), indicating that high Cl^–^ accumulation could prevent the diversion of important nutrients such as NO_3_^–^ or malate. In conditions of low Cl^–^, osmoregulatory and charge balance functions, more importantly occurring at the vacuole, should be compensated by compartmentalization of other molecules (also involved in osmoregulatory functions in plant cells), typically NO_3_^–^ and malate. Once stored in the vacuole, these important sources of N and C are less available for cell metabolism. This suggestion is clearly supported by our results (see Supplementary Table S5 at *JXB* online), showing that leaf NO_3_^–^ concentration was reduced >30-fold in plants when Cl^–^ was increased from the basal (BS) concentration to 5mM Cl^–^, giving rise to a reduction from 49.30mM to 1.52mM NO_3_^–^. Leaf NO_3_^–^ concentration due to the SP treatment was reduced only 3.49-fold when SO_4_^2–^+PO_4_^3–^ was increased from the basal concentration to 5mM SO_4_^2–^+PO_4_^3–^, giving rise to a reduction from 49.30mM to 14.21mM NO_3_^–^. Therefore, the reduction in leaf NO_3_^–^ concentration caused by the CL treatment was 9.35-fold greater than the reduction in leaf NO_3_^–^ concentration caused by the SP treatment. The antagonism between Cl^–^ and NO_3_^–^ accumulation has been widely described and has been attributed to Cl^–^ and NO_3_^–^ competition for plant root uptake (Xu *et al.*, 2000, and references therein). We cannot accept, however, that NO_3_^–^ uptake is reduced by 10-fold in CL plants compared with SP plants given that NO_3_^–^ is the only source of nitrogen used in the nutrient media. This postulate is inconsistent with the increased biomass of CL plants relative to SP plants. Our data clearly support the NO_3_^–^ diversion hypothesis by which macronutrient accumulation of Cl^–^ prevents the use of NO_3_^–^ as an osmolyte or charge-balancing molecule, facilitating its assimilation and increasing plant biomass. Although Cl^–^ has been reported to be interchangeable by NO_3_^–^ (Fricke *et al.*, 1994), we propose that plants preferentially use Cl^–^ for osmoregulatory purposes, while NO_3_^–^, an essential nitrogen source for land plants, is used as an osmolyte when Cl^–^ is not sufficiently available in the soil, or in response to high external NO_3_^–^ concentrations (Siddiq *et al.*, 1991; Radcliffe *et al.*, 2005). This would represent a radical change in the perception of Cl^–^, from an NO_3_^–^ antagonist to a nutrient that promotes a more efficient use of nitrogen. We are currently quantifying the role of Cl^–^ in regulating nitrogen-use efficiency in plants.

Similarly, enhanced Cl^–^ concentrations in leaves could also reduce accumulation of organic anions such as malate, as suggested by the asymmetry between the concentration of inorganic cations and anions (Tables 2, 3). For example, the CL treatment contributes twice as much as the N treatment to achieve electrical balance between cations and inorganic ions (Table 3). From our results we have estimated that the contribution of organic anions to balance the positive charges of inorganic cations is ~65% in N-treated plants, 67% in SP-treated plants, and 25% in CL-treated plants (Table 2). Considering the concentration of the most abundant inorganic ions measured in bulk-mature leaf tissue of plants treated with Cl^–^, an estimation of osmotic potential is obtained (see Supplementary Table S7 at *JXB* online), which corresponds to –1.10MPa. This value is less than the –1.65MPa measured for the plants in the CL treatment (Fig. 5A). This difference of –0.55MPa, accounting for 33% of the osmotic potential, could have been made up by organic and neutral solutes as estimated previously (Tables 2, 3). In other plant species, the contribution of inorganic/organic solutes quantified experimentally in non-stressed plants was similar: 63%/37% in potato (Steward, 1986); 65%/35% in cotton (Meloni *et al.*, 2001); and 77%/23% in wheat (Kameli and Lösel, 1995). Supporting the role of Cl^–^ in preventing diversion of important metabolites, it has been reported that halophyte plants growing under Cl^–^-deficient conditions accumulate higher concentrations of NO_3_^–^ (Neales and Sharkey, 1981; Yeo and Flowers, 1986) and malate (Flowers and Hall, 1978) than those plants growing in optimal NaCl concentrations.

Regarding the photosynthetic metabolism, it should be expected that a significant reduction of *g*_s_ in CL plants (Fig. 6B; Supplementary Figs S4C, S5B at *JXB* online) gave rise to a reduction of *A*_N_. However, *A*_N_ of CL plants was maintained similar to values of BS (Fig. 1A) and SP (Supplementary Figs S4B, S5A) plants. This could be a consequence of a higher diffusion conductance to carbon dioxide (CO_2_) in the leaf mesophyll of CL plants, probably as a consequence of anatomical alterations induced by Cl^–^ in leaf tissues (Fig. 3; Supplementary Fig. S5F). We are presently studying this phenomenon.

### Effect of Cl^–^ on osmotic properties and leaf cell size

It is generally assumed that Cl^–^ is serving a non-specific osmotic function and that other inorganic anions can provide osmolarity to the plant vacuoles or balance positive charges. NO_3_^–^, for example, can certainly do so in the stomata (Geiger *et al.*, 2011). However, this work shows evidence indicating that Cl^–^ is quantitatively and qualitatively a superior osmolyte in plants. Quantitatively, Table 1 shows that Cl^–^, which is not assimilated throughout anabolic metabolism, is accumulated in leaf tissues to a higher concentration than anionic macronutrients such as NO_3_^–^, SO_4_^2–^, or PO_4_^3–^ (Table 1). Calculating the accumulation efficiency of ions according to the molar concentration accumulated in the bulk leaf extract versus the molar concentration applied (see Supplementary Table S6 at *JXB* online), it is observed that the accumulation efficiency of Cl^–^ is comparable with that of potassium, while NO_3_^–^, SO_4_^2–^, and PO_4_^3–^ exhibited lower values. These latter three anionic macronutrients are assimilated in plant metabolism, reducing their internal concentration and therefore their accumulation efficiency. Compared with Cl^–^, NO_3_^–^ accumulation efficiency was about four times lower and that of SO_4_^2–^+PO_4_^3–^ was about three times lower.

Cl^–^ was specifically required to stimulate leaf cell size (Fig. 3). Stimulation of cell size occurred with Cl^–^ treatments as low as 150 μM (Fig. 3H), which represents an increase in the leaf Cl^–^ concentration from 2.49mM (in BS plants) to 6.55mM (in CL plants treated with 150 μM Cl^–^; see Supplementary Table S5 at *JXB* online). This concentration increment is not important enough to play a relevant osmoregulatory role. In the same tissues, SO_4_^2–^+PO_4_^3–^ and NO_3_^–^ concentrations were much higher (26.23mM and 16.30mM, respectively; Supplementary Table S5), suggesting that Cl^–^ plays a specific signalling role in the stimulation of leaf cell growth. With increasing Cl^–^ treatments, a growing response was further observed (Fig. 3H). At least in the millimolar treatment range, this response could be due to a progressive increase in leaf turgor, significantly higher in plants treated with 5mM Cl^–^ (Fig. 5C, F). This could be a consequence of the specific stimulation by Cl^–^ of the tonoplast ATPase (Churchill and Sze, 1984), which favours the vacuolar compartmentalization of Cl^–^, resulting in a more negative osmotic potential and, consequently, a higher turgor than the equivalent SP and N treatments (Fig. 5). Taking both factors together, larger and more turgid cells give rise to leaf tissues with higher capacity for water accumulation (Fig. 4), which is also evidenced by greater leaf thickness (Fig. 3F) and succulence (Fig. 4D) of CL-treated plants. Previous studies have shown that WUE is related to the morphological characteristics of leaves. Wright *et al.* (1994) proposed that there is a close negative relationship between WUE and specific leaf area (SLA), indicating a direct correlation between WUE and leaf thickness (Liu and Stützel, 2004). This correlation was observed in CL-treated plants, but not in SP-treated plants (Supplementary Fig. S7), indicating a relationship between leaf morphological changes and the higher WUE. In addition, the stability of the interaction of water molecules is atypically high in the solvation shell of halogen anions (Kropman and Bakker, 2001), making Cl^–^ an osmolyte with uncommon physical properties, very suitable to stimulate the retention of water in leaf tissue. As a result of the above, Cl^–^ was clearly more efficient in providing leaf osmotic potential, turgor, and water accumulation capacity (Fig. 5), leading to a greater hydration of leaves (Fig. 4).

Specific roles for Cl^–^ in osmotic and cell volume regulation in plants have been previously reported for specialized motor organs or cell types such as the coleoptile of grass seedlings (Babourina *et al.*, 1998), the stigma of grasses at the onset of flower anthesis (Heslop-Harrison and Reger, 1986), the pulvini of *Mimosa pudica* and *Phaseolus vulgaris* during seismonastic leaf movement (Leigh and Wyn Jones, 1985; Fromm and Eschrich, 1988; Iino *et al.*, 2001), epidermal cells from elongating internodes of *Pisum sativum* (Yamagami *et al.*, 2004), and guard cells (Broadley *et al.*, 2012a). Most of these processes have been proved to be responsive to auxin, which stimulates cell Cl^–^ uptake (Babourina *et al.*, 1998; Iino *et al.*, 2001; Yamagami *et al.*, 2004) as a possible prerequisite for cell elongation. We have characterized a Cl^–^ transporter (*At*CCC) strongly expressed in organs involved in primary auxin production (Colmenero-Flores *et al.*, 2007). Lack of function of the *At*CCC gene produces frequent collapse of the elongation zone of the inflorescence stem, possibly due to a failure in the synchronization of physical cell growth and osmolyte (or water) supply during the very fast elongation process of Arabidopsis flowering stems (Colmenero-Flores *et al.*, 2007). These earlier results and the sensitive response of leaf cell size to Cl^–^ shown in this study (Fig. 3) suggest a cross-talk between Cl^–^ nutrition and auxin activity. In marine organisms, animals, and halophyte plants, Cl^–^ plays key roles in cell osmotic, hydric, and turgor (or cell volume) regulation (Flowers, 1988; Sardini *et al.*, 2006; Colmenero-Flores *et al.*, 2007). Through changes in the rate of shoot Cl^–^ buildup, plants might sense Cl^–^ availability and therefore stimulate cell growth to favour more effective compartmentalization of ions and higher water accumulation capacity in plants when such ‘high quality’ electrolyte is accessible. This hypothesis requires a positive feedback regulation mechanism by which Cl^–^ could stimulate the synthesis or stability of auxin. Such a regulatory mechanism has been described in some legumes, where the covalent interaction of chlorine/Cl^–^ with auxin [e.g. indole acetic acid (IAA)] produces a chlorinated auxin (e.g. 4-Cl-IAA) much more active than the non-chlorinated molecule (Böttger *et al.*, 1978; Reinecke, 1999). Under this hypothetical regulatory model, Cl^–^ first induces higher auxin activity, which in turn stimulates both Cl^–^ uptake and cell elongation; two processes that we believe are interconnected. Larger leaf cells with higher ability for ion compartmentalization result in increased water storage capacity. In addition, larger cells also resulted in greater leaf area (Fig. 2C, G), which is expected to determine more light interception and growth. This may also explain why plants treated with Cl^–^ have greater dry biomass (Fig. 1D, F).

### Effect of Cl^–^ on whole-plant water relations

An additional and unexpected effect of Cl^–^ nutrition on the physiology of tobacco plants was a reduction of leaf transpiration (Fig. 6A) as a consequence of the lower stomatal conductance of CL-treated plants (Fig. 6B; Supplementary Figs S4C, S5B at *JXB* online). This phenomenon could be a consequence of: (i) lower stomatal opening; (ii) lower stomatal density; of (iii) lower stomatal index (percentage of stomata out of the total number of epidermal cells plus stomata). The role of anion transport in the regulation of stomatal function is well known. In particular, SLAC1 and SLAH3 channels, which mediate Cl^–^ and NO_3_^–^ release from guard cells, are the components that connect the signalling of environmental cues with the physical events that trigger stomatal closure (Negi *et al.*, 2008; Vahisalu *et al.*, 2008; Geiger *et al.*, 2011). Elevated Cl^–^ content in leaf tissues might alter the flow of anions of epidermal cells, for example due to high extracellular accumulation of Cl^–^, thus modifying the regulatory properties of stomatal activity. On the other hand, specific induction by Cl^–^ of larger epidermal cell size may alter the distribution of the stomata on the leaf surface. Stomatal density, or the number of stomata per unit area, is a function of cell size (Salisbury, 1927). It is expected that an increase in size of epidermal cells, including guard cells, determines a lower stomatal density, which may reduce stomatal conductance per unit leaf area. Although the stomatal index is independent of cell size, an effect of Cl^–^ on the stomatal index cannot be ruled out. We are currently quantifying these anatomical parameters to determine the link between leaf Cl^–^ content and the reduction of stomatal conductance.

### Cl^–^ as a beneficial macronutrient

Cl^–^ is an essential micronutrient (Broyer *et al.*, 1954). The average concentration of Cl^–^ required to meet essential functions as a micronutrient and to determine adequate plant growth is 3.0 μmol g^-1^ DW (Kirkby and Marschner, 2012). We have demonstrated here that Cl^–^ further stimulates tobacco plant growth when accumulated at a concentration 500 times higher (~1,500 μmol g^-1^ DW; Table 1), tissue levels typical of a macronutrient such as potassium. This accumulation specifically determines an increase of plant biomass associated with improved leaf water status, greater WUE, and a remarkable water saving capacity. Effects on leaf water balance and plant water relations are a consequence of anatomical and physiological changes including stimulation of leaf cell size, reduction of stomatal conductance, and induction of additional osmolarity and turgor, which are specifically triggered by Cl^–^ and not by essential anionic macronutrients such as NO_3_^–^, SO_4_^2–^, and PO_4_^3–^. Beneficial elements are defined as those elements that stimulate growth, but are not essential or are essential in certain plant species, or under specific conditions (Broadley *et al.*, 2012b). Since Cl^–^ is not an essential macronutrient but it stimulates growth under such conditions, we propose that Cl^–^ be defined as an essential micronutrient and a beneficial macronutrient. We expect that to a greater or lesser extent Cl^–^ plays the same role in other glycophyte plants. Moreover, we suggest that the biological functions regulated by Cl^–^ availability will probably influence adaptive mechanisms that regulate water homeostasis and the ability of plants to withstand water deficit, a hypothesis that we are currently exploring.

## Supplementary data

Supplementary data are available at *JXB* online

Table S1. Relationship of experiments and figures/tables.

Table S2. Relationship of nutritional treatments.

Table S3. Ion concentration in leaves subjected to different treatments (mg g^-1^ DW).

Table S4. Cl^–^ concentration in different tobacco plant organs (mmol g^-1^ DW).

Table S5. Cl^–^, SO_4_^2–^, PO_3_^3–^, and NO_3_^–^ concentration (mM) in bulk leaf tissues in response to increasing anion concentrations in CL and SP treatment (mM).

Table S6. Accumulation efficiency of nutrients.

Table S7. Osmotic potential calculated from ion concentration measured in mature leaves of 5mM chloride-treated plants.

Figure S1. Cl^–^ deficiency threshold in low Cl^–^ treatments.

Figure S2. Effect of Cl^–^ nutrition on growth parameters.

Figure S3. Effect of Cl^–^ nutrition on plant organ development.

Fig. S4. Efficiency of photosystem II in treated plants.

Figure S5. Complements of Figs 1, 3, and 6.

Figure S6. Identification of CL and SP treatments leading to similar internal concentrations of Cl^–^ and SO_4_^2–^ + PO_4_^3-^ anions.

Figure S7. Effect of Cl^–^ on the relationship between WUE and SLA.

Supplementary Data
