# Supplementary material for: Chloride regulates leaf cell size and water relations in tobacco plants
Source: J Exp Bot. 2015 Nov 23;67(3):873–91. doi: 10.1093/jxb/erv502 (PMC4737079; doi:10.1093/jxb/erv502)
Supplement: Supplementary Data [file supp_67_3_873__index.html]

Chloride regulates leaf cell size and water relations in tobacco plants — Chloride regulates leaf cell size and water relations in tobacco plants — Supplementary Data 

# Chloride regulates leaf cell size and water relations in tobacco plants

## Supplementary Data

Data files

- Supplementary\_figures\_S1\_S7\_\_\_Tables\_S1\_S7.pdf - Supplementary Data
