# Supplementary material for: Chloride regulates leaf cell size and water relations in tobacco plants
Source: J Exp Bot. 2015 Nov 23;67(3):873–91. doi: 10.1093/jxb/erv502 (PMC4737079; doi:10.1093/jxb/erv502)
Supplement: Supplementary Data [file supp_erv502_Supplementary_figures_S1_S7___Tables_S1_S7.pdf]

Table S1. Relation of experiments and figures/tables

	Experiments				
	**JF2012**	**MA2012**	**ND2012**	**AM2013**	**SO2013**
**Fig. 1**	A - D			E - F	
**Fig. 2**	A - D			E - H	
**Fig. 3**	G	A - F		H	
**Fig. 4**	A, C - E			B	
**Fig. 5**	A – F			G	
**Fig. 6**	A - C, E	D			
**Fig. 7**				A – D	
**Fig. 8**				A – D	
**Fig. 9**			All of them		
**Fig. S1**			All of them		
**Fig. S2**	A – B	C – D	E – F		
**Fig. S3**	A – C				
**Fig. S4**			A – D		
**Fig. S5**	D	A – C		F	E
**Fig. S6**				Fig. S6	
**Fig. S7**				Fig. S7	
**Table 1**			All of them		
**Table 2**			All of them		
**Table 3**			All of them		
**Table 4**				Table 4	
**Table 5**				Table 5	
**Table S1**			All of them		
**Table S2**			All of them		
**Table S3**			All of them		
**Table S4**			All of them		
**Table S5**				Table S5	
**Table S6**			All of them		

Table S2. Relation of nutritional treatments

Treatment	Concentration in the irrigation treatment (mM)							
	Cl^-	NO_3^-	SO_4^{2-}	PO_4^{3-}	SO_4^{2-} + PO_4^{3-}	K^+	Ca^{2+}	Mg^{2+}
BS	0.075	5.25	1.140	0.798	1.938	2.12	2.00	1.00
SP 5 mM	0.075	5.25	3.015	2.048	5.063	4.62	2.63	1.63
N 5 mM	0.075	10.25	1.140	0.798	1.938	4.62	2.63	1.63
Cl 5 mM	5.075	5.25	1.140	0.798	1.938	4.62	2.63	1.63
SP 0.15 mM	0.075	5.25	1.190	0.840	2.030	2.19	2.02	1.02
SP 0.30 mM	0.075	5.25	1.250	0.870	2.120	2.27	2.04	1.04
SP 1 mM	0.075	5.25	1.510	1.050	2.560	2.62	2.13	1.13
SP 2.5 mM	0.075	5.25	2.080	1.420	3.500	3.37	2.31	1.31
SP 5 mM	0.075	5.25	3.015	2.048	5.063	4.62	2.63	1.63
CL 0.15 mM	0.151	5.25	1.140	0.798	1.938	2.19	2.02	1.02
CL 0.30 mM	0.301	5.25	1.140	0.798	1.938	2.27	2.04	1.04
CL 1 mM	1.075	5.25	1.140	0.798	1.938	2.62	2.13	1.13
CL 2.5 mM	2.575	5.25	1.140	0.798	1.938	3.37	2.31	1.31
CL 5 mM	5.075	5.25	1.140	0.798	1.938	4.62	2.63	1.63

In most experiments (**JF2012, MA2012, ND2012 & SO2013**) the nutritional treatments were: the basal nutrient solution (BS) alone or supplemented with 5 mM chloride (CL), 5 mM nitrate (N) or the sulphate + phosphate (SP) salt mixture containing the same cationic balance as in the CL and N supplements. In the experiment **AM2013**, BS (0.075 mM Cl^-) is supplemented with 0.15, 0.3, 1, 2.5 or 5 mM chloride (CL), and alternatively the same increasing concentrations with the sulphate + phosphate (SP) salt mixture containing the same cationic balance as in the CL supplements. Red text indicates anions, and blue text indicates cations. Coloured boxes lines denote the concentration of the main anion(s) in each treatment.

Table S3. Ion concentration in leaves subjected to different treatments (mg g^{-1} DW)

**Treatment**	**Ion concentration (mg g^{-1} DW)**						
	**K^+**	**Ca^{2+}**	**Mg^{2+}**	**Cl^-**	**NO_3^-**	**PO_4^{3-}**	**SO_4^{2-}**
**BS**	35.37 **b** ± 1.83	15.68 ± 3.13	6.14 ± 0.51	1.00 **b** ± 0.10	15.82 **b** ± 3.80	12.16 **a** ± 0.23	12.12 **b** ± 0.13
**SP**	48.41 **a** ± 2.32	14.25 ± 1.34	8.16 ± 1.11	0.55 **b** ± 0.06	8.91 **bc** ± 1.75	12.95 **a** ± 0.76	32.29 **a** ± 0.34
**N**	49.24 **a** ± 2.36	18.63 ± 0.63	7.41 ± 0.80	0.59 **b** ± 0.06	38.90 **a** ± 6.30	7.74 **b** ± 0.53	10.13 **b** ± 0.11
**CL**	49.56 **a** ± 1.84	15.29 ± 2.16	7.69 ± 0.77	51.08 **a** ± 2.16	2.45 **c** ± 0.41	9.87 **b** ± 0.41	10.64 **b** ± 0.11
***P*-value**	***	**ns**	**ns**	***	***	***	***

Treatments consisted of the basal nutrient solution (BS) alone or supplemented with 5 mM chloride (CL), 5 mM nitrate (N) or the sulphate + phosphate (SP) salt mixture containing the same cationic balance as in the CL and N supplements. Mean values ± SE, n=6. Levels of significance: $P > 0.05$ ('ns', Not significant differences); $P \leq 0.001$ (***). "Homogeneous group" statistics was calculated through ANOVA.

Table S4. Cl^- concentration in different tobacco plant organs (mmol g^{-1} DW)

Organs	Treatment			
	BS	SP	N	CL
**Roots**	0.0079 **b** ± 0.0001	0.0076 **b** ± 0.0005	0.0960 ± 0.0010	0.2994 **b** ± 0.0118
**Stem**	0.0087 **b** ± 0.0003	0.0102 **ab** ± 0.0009	0.0100 ± 0.0008	0.3880 **b** ± 0.0150
**Leaves**	0.0127 **a** ± 0.0013	0.0122 **a** ± 0.0008	0.0109 ± 0.0004	1.2873 **a** ± 0.0510
***P*-value**	**	***	**ns**	***

Treatments consisted of the basal nutrient solution (BS) alone or supplemented with 5 mM chloride (CL), 5 mM nitrate (N) or the sulphate + phosphate (SP) salt mixture containing the same cationic balance as in the CL and N supplements. Mean values ± SE, n=6. Levels of significance: $P > 0.05$ ('ns', Not significant differences); $P \leq 0.01$ (**); $P \leq 0.001$ (***). "Homogeneous group" statistics was calculated through ANOVA.

Table S5. Cl^-, SO_4^{2-}, PO_3^{3-} and NO_3^- concentration (mM) in bulk leaf tissues in response to increasing anions concentrations in CL and SP treatment (mM)

Anion	ID	Treatment (mM)								
		Anova *P*-value	0.075 (BS)	0.150	0.300	1.0	2.5	5.0	Manova *P*-value	
Cl^-	SP	*	2.49 **ab** ± 0.14	2.80 **a** ± 0.48	1.82 **ab** ± 0.39	1.57 **ab** ± 0.28	1.39 **b** ± 0.23	1.27 **b** ± 0.22	**b**	***
	CL	***	2.49 **e** ± 0.14	6.55 **de** ± 1.00	13.44 **d** ± 0.71	35.82 **c** ± 1.52	75.11 **b** ± 1.70	104.09 **a** ± 3.17	**a**	
SO_4^{2-} + PO_4^{3-}	SP	***	24.13 **b** ± 3.07	24.19 **b** ± 2.99	26.16 **b** ± 3.51	26.97 **b** ± 1.99	33.66 **ab** ± 3.72	48.02 **a** ± 4.25	**a**	***
	CL	**	24.13 **ab** ± 3.07	26.23 **a** ± 2.99	19.67 **ab** ± 1.16	16.27 **b** ± 1.72	16.90 **b** ± 0.29	16.17 **b** ± 0.67	**b**	
SO_4^{2-}	SP	***	13.98 **b** ± 2.74	14.16 **b** ± 2.95	15.78 **b** ± 3.24	16.59 **b** ± 2.31	23.58 **ab** ± 3.58	36.40 **a** ± 3.94	**a**	***
	CL	**ns**	13.98 ± 2.74	15.68 ± 3.01	10.31 ± 1.22	9.19 ± 1.43	8.20 ± 0.28	8.30 ± 0.63	**b**	
PO_4^{3-}	SP	**ns**	10.15 ± 0.46	10.03 ± 0.65	10.36 ± 0.33	10.36 ± 0.24	10.07 ± 0.19	11.61 ± 0.50	**a**	***
	CL	***	10.15 **a** ± 0.46	10.55 **a** ± 0.68	9.36 **ab** ± 0.62	7.08 **c** ± 0.47	8.71 **abc** ± 0.24	7.87 **bc** ± 0.18	**b**	
NO_3^-	SP	*	49.30 **a** ± 9.16	35.31 **ab** ± 4.00	26.61 **ab** ± 7.02	20.96 **ab** ± 4.99	7.56 **b** ± 2.21	14.21 **b** ± 9.33	**a**	***
	CL	**	49.30 **a** ± 9.16	16.30 **b** ± 2.74	15.47 **b** ± 3.57	7.89 **bc** ± 3.50	5.14 **bc** ± 2.04	1.52 **c** ± 0.49	**b**	

The basal nutrient solution (BS, 0.075 mM Cl^-) is supplemented with 0.150, 0.300, 1.0, 2.5 of 5 mM chloride (CL), and alternatively the same increasing concentrations with the sulphate + phosphate (SP) salt mixture containing the same cationic balance as in the CL supplements. ANOVA statistical test compared over increasing concentrations within each treatment (SP or CL); and MANOVA statistical test compares the tendency of both treatments throughout growing concentrations. Mean values ± SE, n=6. Levels of significance: $P > 0.05$ ('ns', Not Significant differences); $P \leq 0.05$ (*); $P \leq 0.01$ (**); and $P \leq 0.001$ (***).

Table S6. Accumulation efficiency of nutrients

Ion	Treatment			
	**BS**	**SP**	**N**	**CL**
**K^+**	39.39 **a** ± 2.12	25.01 **a** ± 2.12	26.49 **a** ± 2.48	24.37 **a** ± 1.42
**Cl^-**	38.02 **b** ± 4.44	18.10 **b** ± 1.67	21.40 **b** ± 2.39	24.34 **a** ± 1.23
**NO_3^-**	3.99 **c** ± 0.94	2.61 **d** ± 0.52	6.29 **c** ± 1.22	0.67 **c** ± 0.12
**$SO_4^{2-} + PO_4^{3-}$**	11.12 **c** ± 0.85	8.09 **c** ± 0.89	7.30 b**c** ± 0.27	8.93 **b** ± 0.45
***P*-value**	***	***	***	***

Ion accumulation efficiency was calculated according to the molar concentration accumulated in the bulk leaf extract vs. the molar concentration applied. Mean values ± SE, n=6. Levels of significance: $P \leq 0.001$ (***). "Homogeneous group" statistics was calculated through ANOVA.

Table S7. Osmotic potential calculated from ion concentration measured in mature leaves of 5 mM chloride-treated plants.

**Ions**	**MW (g mol^{-1})**	**mM**	**-MPa**
**K^+**	39.10	133	-0.340
**Na^+**	22.99	16	-0.069
**Ca^{2+}**	40.08	40	-0.100
**Mg^{2+}**	24.30	33	-0.136
**Cl^-**	35.45	151	-0.426
**NO_3^-**	61.97	4	-0.006
**SO_4^{2-}**	96.01	11	-0.011
**PO_4^{3-}**	94.93	11	-0.012
**Ψπ**			-1.10
**Ψπ (Fig. 5A)**			-1.65
**Missing Ψπ**			-0.55

Osmotic potential (Ψπ) calculated from the ion concentration obtained in mature leaves of 5 mM Cl^--treated plants (CL) at the same experiment (JF2012) that correspond to the osmotic potential (Ψπ) measured in mature leaves by wescor chamber (Figure 5). Missing osmotic potential is the difference between measured osmotic potential and calculated osmotic potential.

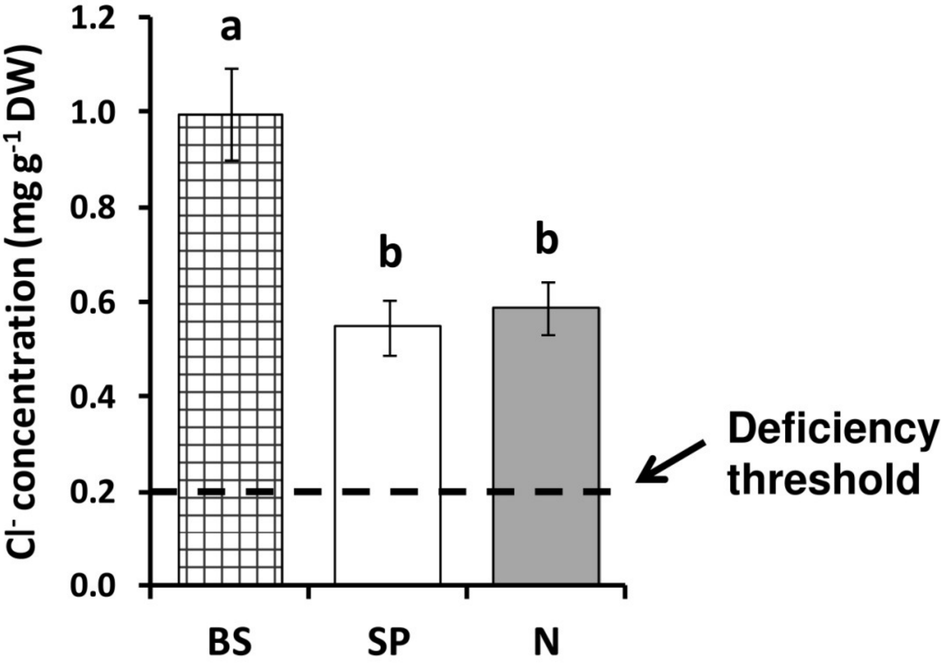

Fig. S1. Cl^- deficiency threshold in low-Cl^- treatments. Treatments consisted of the basal nutrient solution (BS) alone or supplemented with 5 mM nitrate (N) or the sulphate + phosphate (SP) salt mixture containing the same cationic balance as in the CL and N treatments. Deficiency threshold is reported at a concentration of around 0.2 mg g^{-1} DW (reviewed in Flowers, 1988; Xu *et al.*, 2000; White and Broadley, 2001; Broadley *et al.*, 2012a). Mean values ± SE, *n*=6. "Homogeneous group" statistics was calculated through ANOVA test.

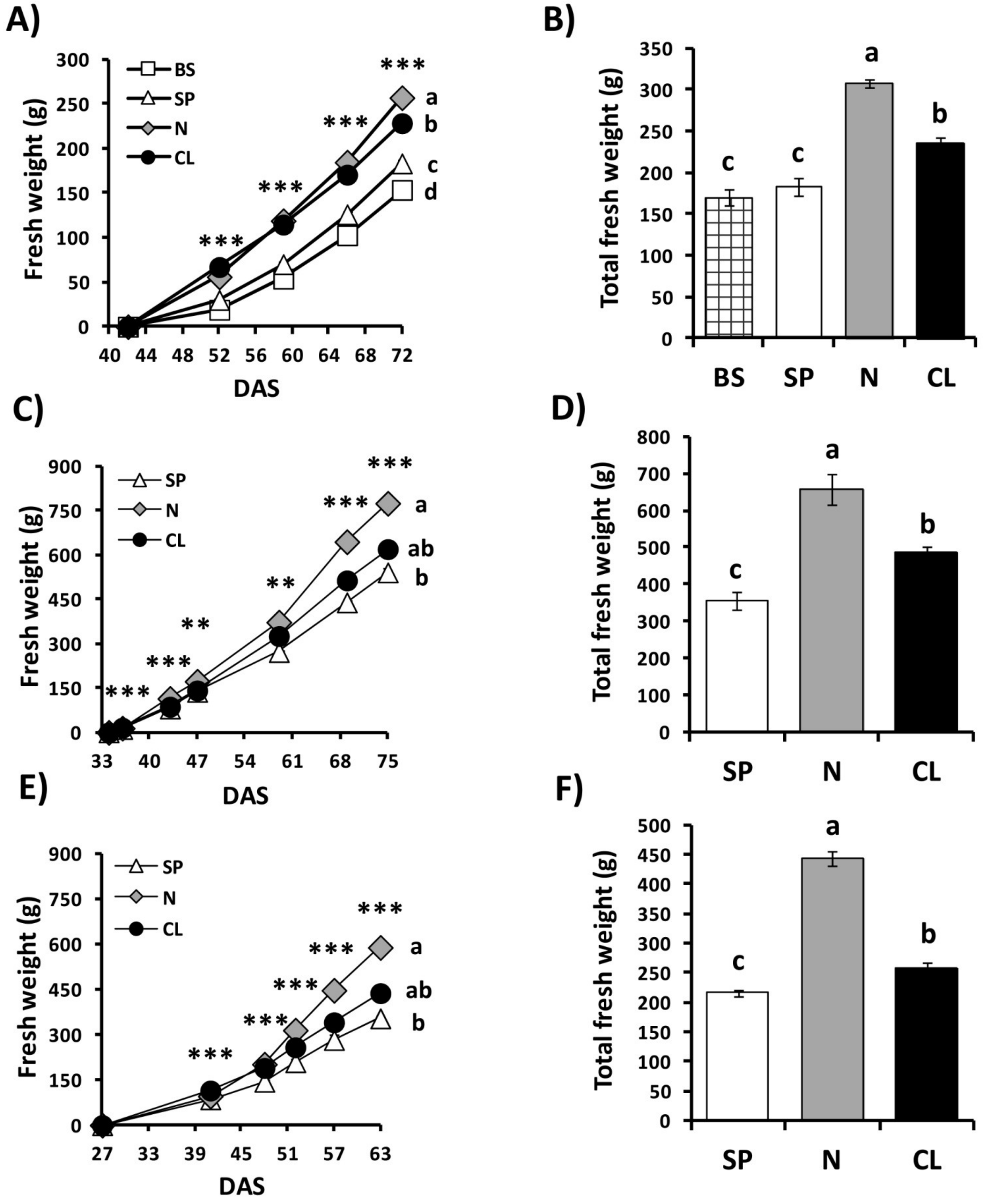

Fig. S2. Effect of Cl^- nutrition on growth parameters. Treatments consisted of the basal nutrient solution supplemented with 5 mM chloride (CL), 5 mM nitrate (N) or the sulphate + phosphate (SP) salt mixture containing the same cationic balance as in the CL and N treatments. (A, C, E) Fresh weight evolution over time of three independent experiments, and (B, D, F) total biomass at the end of each experiment, respectively. DAS, Days After Sowing. Mean values ± SE, *n*=4-6. Levels of significance: $P \leq 0.01$ (**); $P \leq 0.001$ (***); and "homogeneous group" statistics was calculated through ANOVA (A-F) and MANOVA test (A, C, E).

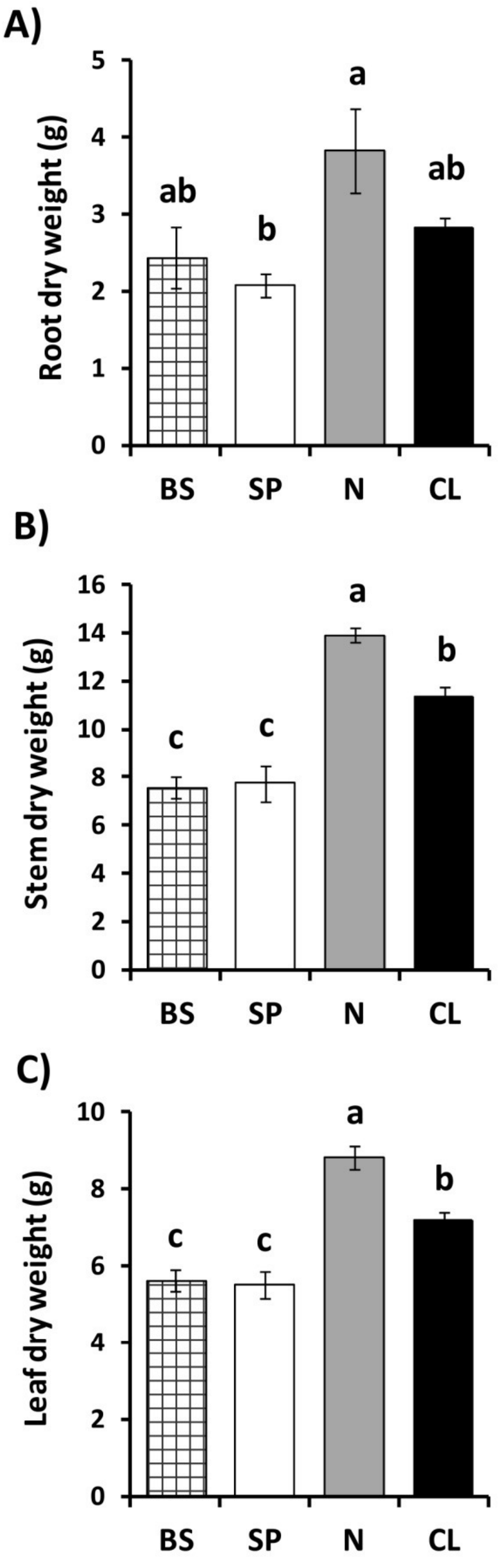

Fig. S3. Effect of Cl^- nutrition on plant organs development. Treatments consisted of the basal nutrient solution (BS) alone or supplemented with 5 mM chloride (CL), 5 mM nitrate (N) or the sulphate + phosphate (SP) salt mixture containing the same cationic balance as in the CL and N treatments. (A) Root dry biomass. (B) Stem dry biomass. (C) Total leaf dry biomass. Mean values ± SE, *n*=4-6. "Homogeneous group" statistics was calculated through ANOVA (A-C).

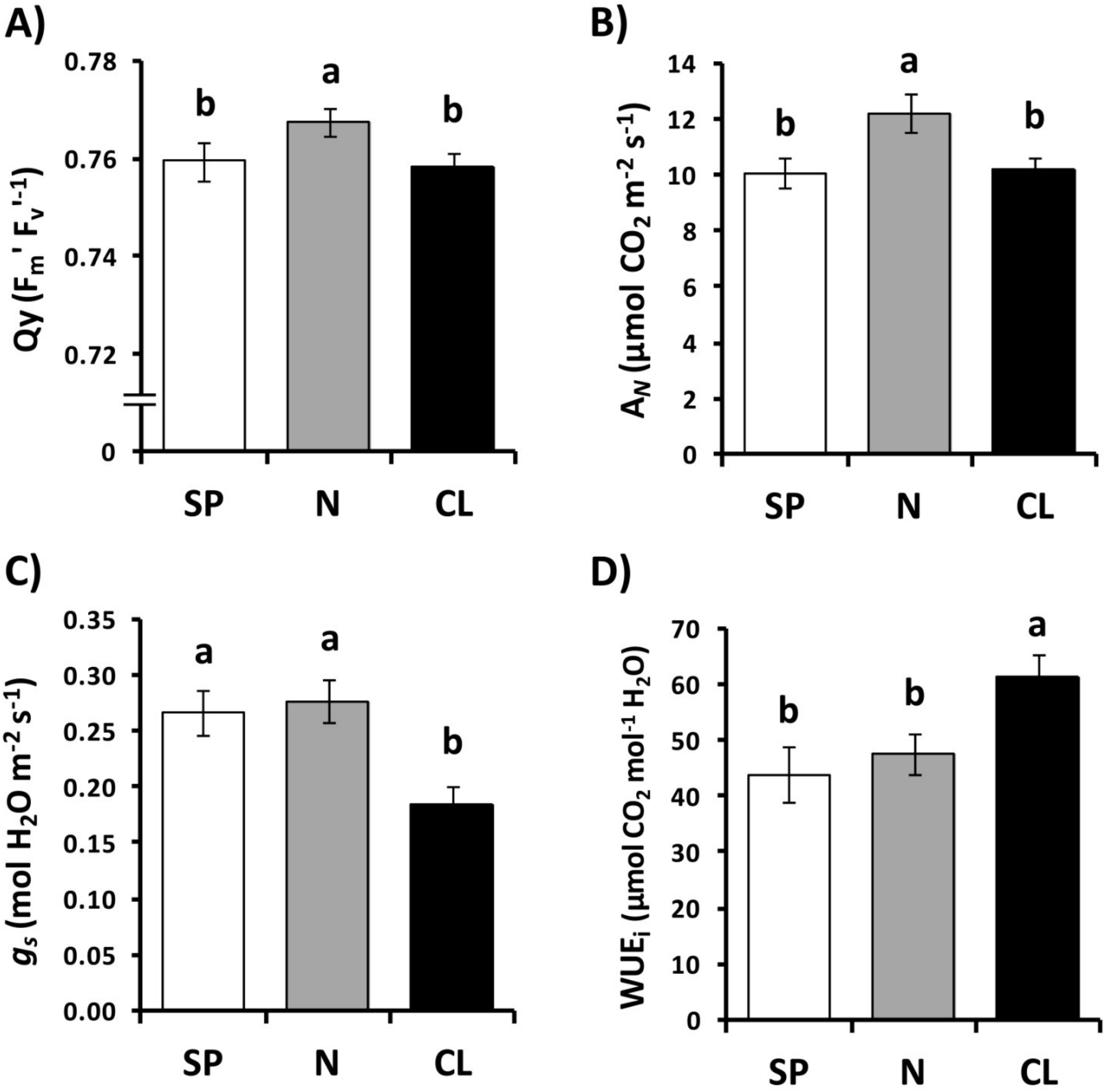

Fig. S4. Efficiency of Photosystem II in treated plants. Treatments consisted of the basal nutrient solution supplemented with 5 mM chloride (CL), 5 mM nitrate (N) or the sulphate + phosphate (SP) salt mixture containing the same cationic balance as in the CL and N treatments. (A) The highly sensitive physiological stress marker quantum yield (Qy) was quantified in the experimental conditions where chloride treatment produced the effects described in this paper. To do this, in the same plants was also quantified: (B) Net photosynthetic rate (A_N); (C) stomatal conductance (g_s); (D) photosynthetic or instantaneous water-use efficiency (WUEi). Photosynthetically active leaves from plants between 68-73 days after sowing (DAS) were used. Mean values ± SE, n=4-6. “Homogeneous group” statistics was calculated through ANOVA test (A-D).

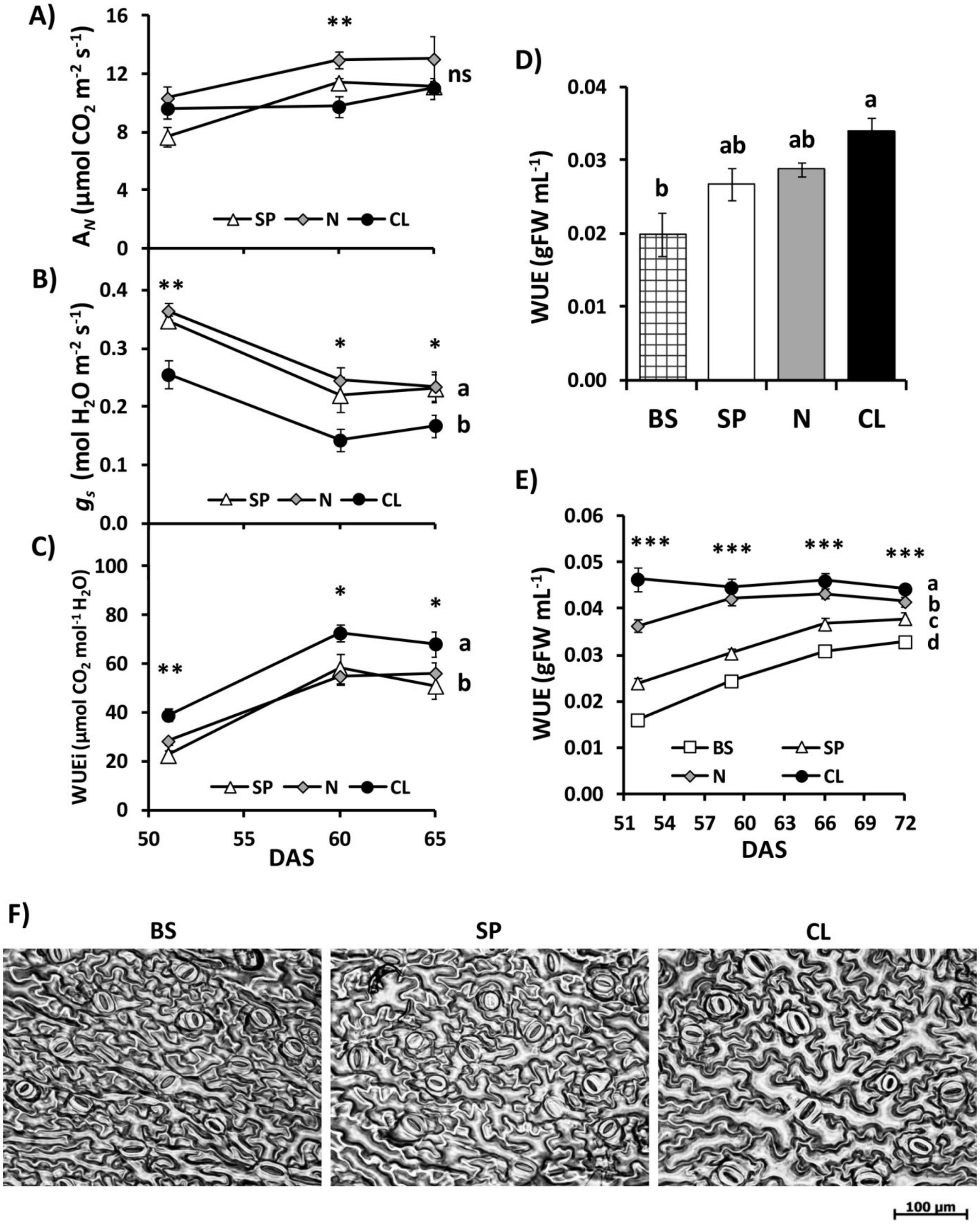

Fig. S5. Complements of figures 1, 3 and 6. Trials are presented to show experimental treatments that are not present in some of the experiments shown in the manuscript. Treatments consisted of the basal nutrient solution (BS) alone or supplemented with 5 mM chloride (CL), 5 mM nitrate (N) or the sulphate + phosphate (SP) salt mixture containing the same cationic balance as in the CL and N treatments. Effect on (A) net photosynthetic rate (A_N), (B) stomatal conductance (g_s) and (C) photosynthetic or instantaneous water-use efficiency (WUEi) measured from plants between 50-65 days after sowing (DAS). (D-E) Effect on integrated water-use efficiency (WUE) obtained in two independent experiments. WUE is calculated as total biomass produced in relation to total water consumed (D) at the end of the experiment or (E) measured from plants between 51-72 days after sowing (DAS). (F) Effect on epidermal cell size observed in microscopy images of abaxial leaf epidermal impressions from the assay AM2013 with the treatments BS, SP and CL. Mean values ± SE, *n*=6. Levels of significance: $P > 0.05$ ('ns', Not significant differences); $P \leq 0.05$ (*); $P \leq 0.01$ (**); $P \leq 0.001$ (***). "Homogeneous group" statistics was calculated through ANOVA (A-E) and MANOVA (A-C, E) tests.

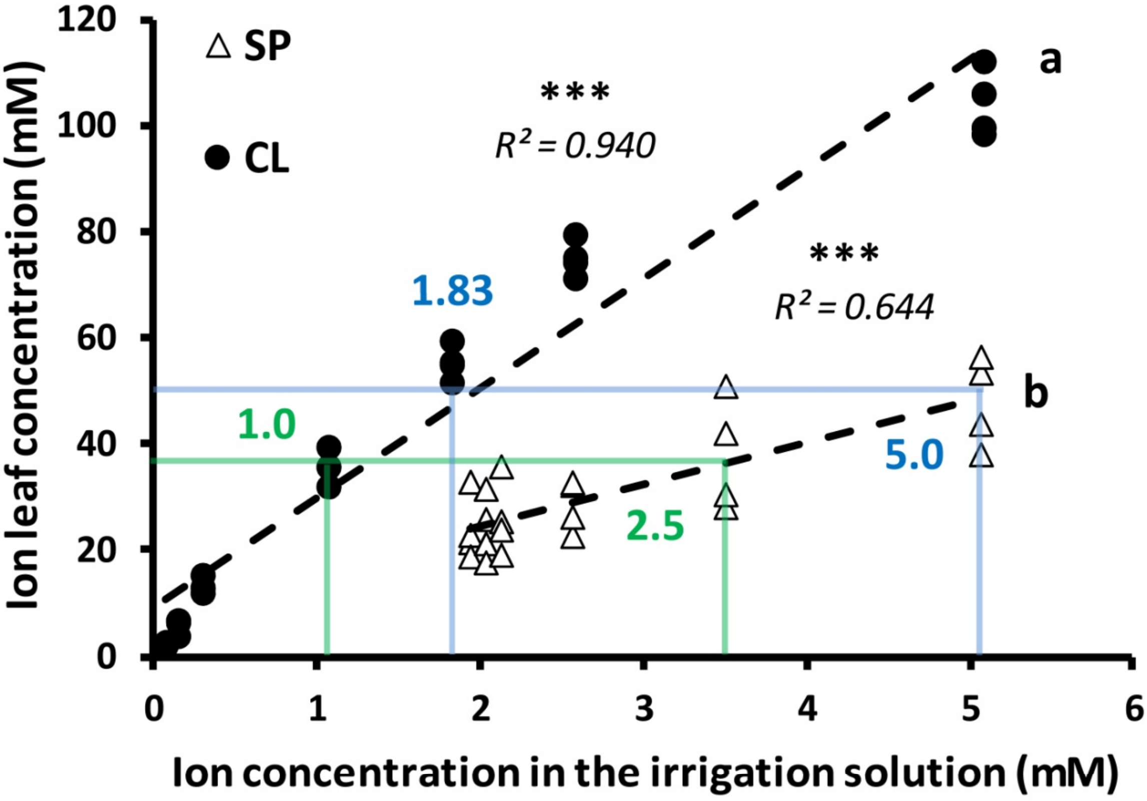

Figure S6. Identification of CL and SP treatments leading to similar internal concentrations of Cl^- and SO_4^{2-} + PO_4^{3-} anions. Increasing concentrations of Cl^- (CL) or sulphate + phosphate (SP) treatments, maintaining the same cationic balance, were use to quantify internal concentrations of Cl^- and SO_4^{2-} + PO_4^{3-} anions. The supplemented treatments are based on a basal irrigation solution (BS) which contains sulphate, phosphate and nitrate because those macronutrients are necessary in millimolar concentration for the proper development of plants. For more information about irrigation solutions see the Supplementary Table S2. We obtained comparable internal concentrations of Cl^- (35.82 ± 1.52 mM) and SO_4^{2-} + PO_4^{3-} (33.65 ± 3.72 mM) in the leaf when the anion concentration in the supplemented irrigation solution was **1** mM Cl^- and **3.5** mM SO_4^{2-} + PO_4^{3-} (1.562 mM SP-treatment + 1.938 mM SO_4^{2-} and PO_4^{3-} already present in the basal solution) respectively. Comparable internal concentrations of Cl^- (55.46 ± 7.50 mM) and SO_4^{2-} + PO_4^{3-} (48.01 ± 4.25 mM) were also found in the leaf when the anion concentration in the supplemented irrigation solution was **1.83** mM Cl^- and **5.0** mM SO_4^{2-} + PO_4^{3-} (3.125 mM SP-treatment + 1.938 mM SO_4^{2-} and PO_4^{3-} already present in the basal solution), respectively. Levels of significance represented by the Pearson's *R*-squared linear correlation test (R^2), and $P \leq 0.001$ (***). “Homogeneous group” statistics was calculated through MANOVA tests.

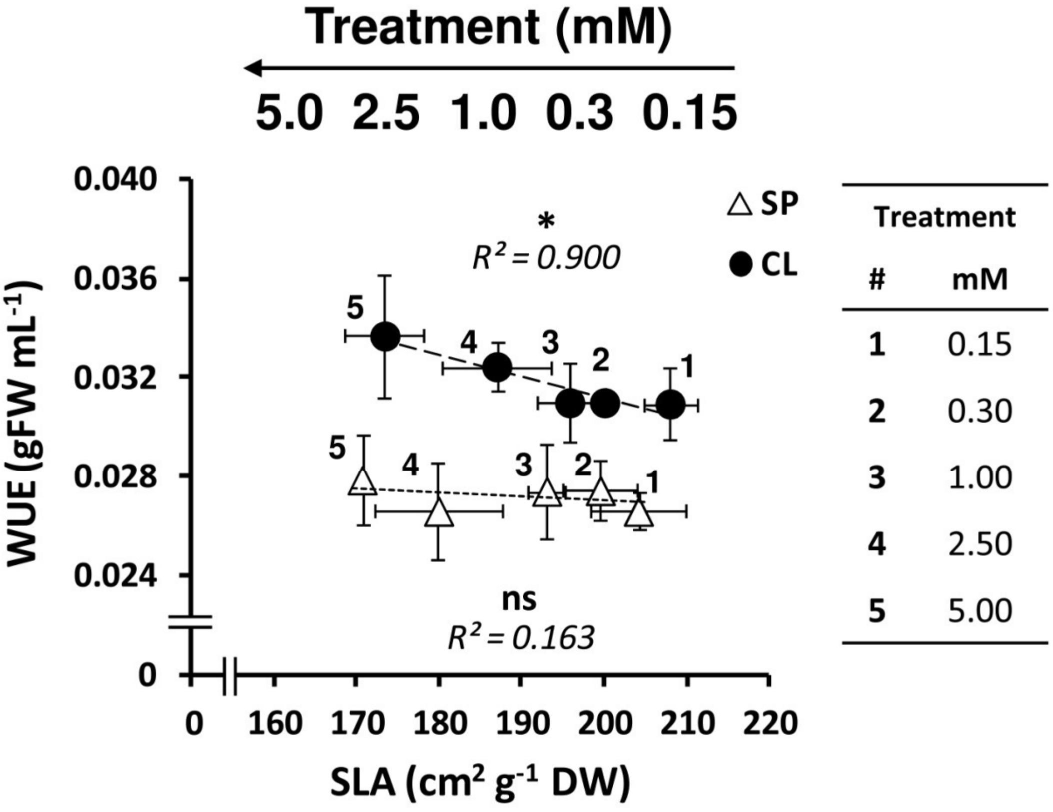

Figure S7. Effect of Cl^- in the relationship between WUE and SLA. Treatments consisted of increasing concentrations of Cl^- (CL) or sulphate + phosphate (SP) salts maintaining the same cationic balance. Correlations with integrated water use efficiency (WUE) to specific leaf area (SLA) measured in both CL and SP plants are given with filled circles and open triangles, respectively. Increasing anion concentration in the irrigation solution from 0.15 mM (1) to 5 mM (5), is schematized over the graph and represented in the table. Mean values ± SE, n=6. Levels of significance represented by the Pearson's R-squared linear correlation test (R^2) and $P > 0.05$ (‘ns’, Not significant differences), and $P \leq 0.05$ (*).
